# Supplementary material for: Does service heterogeneity have an impact on acute hospital length of stay in stroke? A UK-based multicentre prospective cohort study
Source: BMJ Open. 2019 Apr 3;9(4):e024506. doi: 10.1136/bmjopen-2018-024506 (PMC6500188; doi:10.1136/bmjopen-2018-024506)
Supplement: Supplementary data [file bmjopen-2018-024506supp001.pdf]

Myint et al. BMC Health Services Research 2011, 11:50
http://www.biomedcentral.com/1472-6963/11/50

BMC Health Services Research

STUDY PROTOCOL
Open Access

Evaluation of stroke services in Anglia stroke clinical network to examine the variation in acute services and stroke outcomes

Phyo K Myint[1,2*], John F Potter[1,2], Gill M Price[1], Garry R Barton[1], Anthony K Metcalf[2], Rachel Hale[2], Genevieve Dalton[3], Stanley D Musgrave[1], Abraham George[4], Raj Shekhar[5], Peter Owusu-Agyei[6], Kevin Walsh[7], Joseph Ngeh[8], Anne Nicholson[9], Diana J Day[10], Elizabeth A Warburton[10], Max O Bachmann[1]

Abstract

Background: Stroke is the third leading cause of death in developed countries and the leading cause of long-term disability worldwide. A series of national stroke audits in the UK highlighted the differences in stroke care between hospitals. The study aims to describe variation in outcomes following stroke and to identify the characteristics of services that are associated with better outcomes, after accounting for case mix differences and individual prognostic factors.

Methods/Design: We will conduct a cohort study in eight acute NHS trusts within East of England, with at least one year of follow-up after stroke. The study population will be a systematically selected representative sample of patients admitted with stroke during the study period, recruited within each hospital. We will collect individual patient data on prognostic characteristics, health care received, outcomes and costs of care and we will also record relevant characteristics of each provider organisation. The determinants of one year outcome including patient reported outcome will be assessed statistically with proportional hazards regression models. Self (or proxy) completed EuroQol (EQ-5D) questionnaires will measure quality of life at baseline and follow-up for cost utility analyses.

Discussion: This study will provide observational data about health service factors associated with variations in patient outcomes and health care costs following hospital admission for acute stroke. This will form the basis for future RCTs by identifying promising health service interventions, assessing the feasibility of recruiting and following up trial patients, and provide evidence about frequency and variances in outcomes, and intra-cluster correlation of outcomes, for sample size calculations. The results will inform clinicians, public, service providers, commissioners and policy makers to drive further improvement in health services which will bring direct benefit to the patients.

Background

Stroke is the third leading cause of mortality and the number one cause of long-term disability in the UK. More than 150,000 people suffer a stroke in the UK each year [1]. It costs the NHS approximately £ 7 billion per annum [2]. Stroke incidence rises sharply with age and despite better primary and secondary preventative measures, the total number of strokes is set to rise in the UK [3]. Nevertheless, stroke care in UK is far from ideal: patients having a worse outcome in terms of death and dependency than many other European countries [4-6], at least in part due to differences in care provided [7]. There is also variation in outcome between different localities within the UK [8-11], these local differences being highlighted in the most recent publication of the National Sentinel Stroke Audit in 2009 [12]. These differnces probably arise as a result of substantial variations in how the stroke services are provided across the UK. Examples of such differences are access to neurovascular/neurosurgical service, early supported

* Correspondence: phyo.k.myint@uea.ac.uk
[1]Norwich Medical School, Faculty of Medicine & Health Sciences, Norwich, UK
Full list of author information is available at the end of the article

© 2011 Myint et al; licensee BioMed Central Ltd. This is an Open Access article distributed under the terms of the Creative Commons Attribution License (http://creativecommons.org/licenses/by/2.0), which permits unrestricted use, distribution, and reproduction in any medium, provided the original work is properly cited.

discharge, and stroke specialist on call rota for thrombolysis. The presence or absence of variations in stroke outcomes as a result of variation in care and how much the observed variations in patients' outcomes including patient reported outcome measure (PROM) are determined by the differences in service delivery have not been examined previously.

We hypothesise that variation in patient outcomes including mortality, length of stay, institutionalisation rate, and patient reported outcomes between care providers can partly be explained by the different ways in which stroke services are delivered. The main objectives of the study are (1) to describe variation in outcomes following stroke and to identify the characteristics of services that are associated with better outcomes after accounting for case mix differences and individual prognostic factors, and (2) to obtain preliminary data to identify sample size and inform future pragmatic real world setting RCTs in the area of health service delivery in stroke.

Methods/Design

A prospective cohort study will be conducted to identify characteristics of services that are associated with the best outcomes including patient reported outcomes, taking into account case-mix and patients' prognostic features. The study will consist of two components (1) consecutive stroke admissions in selected months (a total of 8 months) and (2) a prospective study of patient reported outcome in some of these selected months.

Sample Population

For the first component, the sample population will be stroke patients who are admitted to any of the hospitals within the Anglia region of Stroke & Heart Clinical Network between October 2009 and September 2011. Baseline data are already recorded, prior to the study commencement, as part of routine clinical data collection by Anglia Stroke Clinical Network (as described in detail below). The study sample will be a systematically selected sample (every third month) rather than a consecutive cohort of patients admitted to eight acute NHS hospital trusts. Therefore, this is not a consecutive case study; instead it seeks to be representative of the catchment population of the hospital and has taken into account the seasonal variation in stroke incidence and outcome [13].

For the patient reported outcome component of the study the following inclusion and exclusion criteria will be used. Inclusion criteria are (1) age > = 18 years, (2) admitted to hospital with stroke (diagnosed by stroke physicians) during the study months, (3) able to provide informed consent or patient's personal consultee agrees to study participation. Exclusion criteria include (1) age <18 years, (2) patients with pre-existing diagnosis of dementia (for PROM component only).

The Anglia Stroke Network was funded through the NHS Improvement Programme, following the publication of the National Stroke Strategy in December 2007. The Network was established in April 2008 to support the development of stroke services in Norfolk, Suffolk and Cambridgeshire regions. Since its inception, the Network regularly collected data to capture clinical service activities of the eight acute hospital trusts in the Network for the purpose of monitoring of services benchmarked by National targets and guidance from National Institute of Health & Clinical Excellence (NICE) in England and Wales. Data collection commenced in January 2009 and involves the individual trusts collecting clinical data which is fed back to the network by monthly reports. The total number of strokes admitted to the 8 acute trusts within the Network is approximately 4,000 per annum in 2009. The stroke cases were identified prospectively data were collected by the clinical team who looked after the patients and anonymised raw clinical data were sent to the network on monthly basis. The network collates and analyses the data for above mentioned purposes.

Sample size

Since this is an exploratory study designed to provide information for further analytic research, sample size will be determined partly pragmatically rather than on particular hypothesis tests. For illustration purposes, a total sample of 2264 patients would provide 80% power to detect a constant Hazard ratio (HR) of 0.76 for one-year mortality between two groups of roughly equal size, based on the log-rank test. This assumes a 20% one-year mortality rate in the reference group, no loss to follow-up before one year and 2-sided type I error of 5%. If one-year mortality is 30%, then 2264 patients would provide 76% power to detect a HR of 0.81.

Plan of investigation

The study will have a cohort design. We will follow up a cohort of patients systematically selected from each trust. For pragmatic purposes we will sample all patients who are admitted every third month, starting from October 2009. Over one calendar month, there will be ~ 300-350 stroke cases entered into the Network Clinical Data. Between October 2009 and September 2011, the Clinical Network would have collected a total of eight 3-monthly datasets per trust (i.e. 8 study months in total: Oct 2009, Jan 2010, April 2010, July 2010, October 2010, Jan 2011, April 2011, July 2011). Therefore, the estimated total cohort size with baseline clinical data will be ~ 2,400 stroke cases

during this exercise (30% of 4000 patients admitted annually in 8 trusts = 1200 × 2 yrs).

We will collect patient data by hospital trusts and conduct a questionnaire survey of patients' outcomes. Due to the nature of the study we would need 100% follow-up in randomly selected populations. Because we will be using a partially historical cohort, to avoid selection bias for mortality outcome, informed consent from all eligible participants will not be feasible. Therefore, it is most appropriate for the clinical team to collect the outcome data to comply with current ethical guidance in the UK. Therefore, the identifiable patient data will only be held at the local NHS trusts.

Neither the network nor the investigators will have access to any identifiable patient information (e.g. name, address). For outcome data we will utilise death certificate and hospital episode data from the Patient Administrative System (PAS) as described previously [14,15]. This approach will be used in conjunction with telephone and postal follow-up for questionnaire surveys such as EQ-5 D, and Stroke Impact Scale. These data will be counter-checked using discharge coding records, which record each hospital episode.

The clinical teams will retrieve case records to collect (1) baseline measures which were not recorded in baseline Network surveys and (2) outcome measures including mortality and hospital length of stay. At study commencement (October 2010) one year follow up data can be collected immediately for October 2009 cohorts (follow up complete at end September 2010). The follow up will be completed in September 2012 as the stroke patients included in the last survey for the study conducted by the Network in July 2011 will complete one year follow-up in June 2012 and data collection of the study will be completed by July-August 2012 with the view of final cohort data arrival to research team by the end of December 2012.

Due to multi-centre nature of the study the individual sites are expected to join the study at different time points (after their respective NHS Research & Development Committees' approval). We will collect characteristics of stroke services, patient related factors, prognostic indicators, treatment options and trial/study participation. Missing prognostic data will be imputed statistically, to ensure that all eligible patients are included in the primary analysis (see also Statistical Methods).

The service characteristics of interest include:

At hospital level

- staffing (including junior doctors and therapists (whole time equivalent), physicians characteristics
- university or district general hospital
- distance from tertiary referral centre
- availability of vascular surgery on site, neuro-surgery and neuro ITU on site
- monitoring beds
- physician on call rota
- compliance with NICE guidelines

At patient level

- provision of thrombolysis and CT
- medication

Outcome measurements

Primary outcome of the study will be one year mortality comparison between services with different characteristics. The secondary outcomes will include (1) final discharge destination (good or poor outcome) [16], (2) length of acute hospital stay, (3) length of stay in rehabilitation, (4) complications during acute and rehab-hospital stay and significant procedures (e.g. aspiration pneumonia, myocardial infarction), (5) readmissions, (6) composite cardiovascular events (recurrent TIA/Stroke/Acute Coronary Syndrome, Myocardial infarction).

Patient Reported Outcome Measures (PROM)

PROM will consist of (1) Stroke Impact Scale, (2) health related quality of life: EQ-5 D at one year in those who completed questionnaire at the baseline, (3) modified RANKIN, (4) Barthel score and (5) health service use.

Statistical analysis

Quantitative data will be analysed by multivariate Cox-proportional hazards to examine the relationships between different aspects of health services and time to death, adjusting for prognostic characteristics. Multiple logistic or linear regression models will be constructed as appropriate for dichotomised and continuous outcome variables respectively. T tests for normally distributed data and Mann-Whitney U tests for non-normally distributed data will be used to compare continuous outcomes. Volume-outcome relationships will be investigated. Missing prognostic and EQ-5 D data will be imputed, based on each patient's other prognostic characteristics. Clustering of data by hospital trust will be investigated and, if necessary, taken into account, and intra-class correlation coefficients calculated to inform future research.

Economic evaluation

Health care resources are scarce and it is therefore important to ensure that evaluations are undertaken in order to ensure that services provided by the NHS constitute value for money. Within this study we will thereby seek to estimate the cost-effectiveness of different stroke service deliveries.

Costs will first be calculated from the perspective of the NHS and personal social services (PSS). Thus, levels

of resources use will be recorded during the follow-up period, including the length of original hospital stay, input by the multi-disciplinary team, other investigations (e.g. x-ray) and any complications (including details of any further hospital admissions). Unit costs will subsequently be assigned to each of these resource items, enabling both the total mean cost in participants and the incremental cost between two different service deliveries (chosen to compare the cost effectiveness, e.g. traditional on call rota vs. telemedicine) to be calculated after adjusting for other factors. The main measure of effectiveness to be used in the economic analysis will the EQ-5 D [17], where responses will be sought at baseline, and at 12 month as mentioned above. This will enable the overall effect of each mode of service delivery, and the incremental effect of services to be estimated.

Outcome

As the National Institute of Health and Clinical Excellence [18] recommends use of the EQ-5 D [17] within cost-effectiveness analysis this will be our primary measure within the economic analyses. EQ-5 D data will be collected at two University Hospitals and two district general hospitals within the clinical network. We will use "mapping" strategy to estimate the cost-effectiveness analyses across the region. The use of mapping, where scores from a condition-specific (non preference-based) measure are 'converted' into a utility (preference-based) score using a pre-defined formulae, has been advocated (in certain instances) by the UK National Institute of Health and Clinical Excellence (NICE) [18], and has been used to estimate the utility scores, and in turn cost-effectiveness, of a number of health care interventions [19]. Mapping presents the possibility of not asking all participants to complete the EQ-5 D. In this study we propose to take advantage of this by developing a mapping algorithm based on the response from participants participating in this component to predict the EQ-5 D for participants in retrospective cohorts and those who did not participate in PROM component.

Because the quality of life measure (EQ-5D) which can be used to estimate health utility and calculate QALYs (Quality Adjusted Life Years) for economic evaluation is outside the remit of routine data collection and cannot be done retrospectively, we will collect EQ-5 D data in only the second year of the study (October 2010 and January, April and July 2011 cohorts and one year follow up data to be collected September and December 2011, and March and June 2012) in those who provide informed consent to the study (we estimate that the sample will be approximately 15-20% of the whole sample after excluding the one year pre-study period (between October 2009-September 2010) and after taking into account of refusal rate (estimated ~ 30%) in trusts with Stroke or Comprehensive Local Research Network Research Nurses.

Economic Analysis

In the Economic analysis if one option is shown to be less costly and more effective than another option (for example, telemedicine vs. on call system) then that option will 'dominate' the other and be deemed cost-effective. Alternatively, the incremental cost-effectiveness ratio (ICER) associated with a particular option will be estimated and assessed in relation to a range of cost-effectiveness thresholds. The associated level of uncertainty will also be characterised by e.g. estimating the cost-effectiveness acceptability curve (CEAC) for each intervention and conducting value of information analysis [20]. Sensitivity analysis will also be undertaken to assess the robustness of conclusions to key assumptions. We will also seek to identify what resource items should be monitored in a future study (i.e. what are the big cost drivers which are likely to be affected by the intervention) and how these items should be identified.

The study is funded by the NIHR Research for Patient Benefit Programme (PB-PG-1208-18240) and obtained ethical approval from the Norfolk Research Ethics Committee.

Discussion

In this study we specifically aim to identify services that are associated with the best clinical outcomes including mortality and hospital length of stay including patient reported outcome adjusting for patient prognostic factors and potential confounders. Our study will be able to provide useful information in stroke service provision in UK and beyond. Furthermore, inclusion of patient reported outcome is novel and exciting component of our study.

Studies which have examined the delivery of specific services such as rapid imaging, have shown improvement in patients' outcome in stroke [21]. A recent report from Germany suggested that a telestroke network may be a useful strategy to implement in their non-urban stroke services [22]. Lees et al (2008) [23] highlighted that there is room for improvement in terms of acute services for stroke. Interestingly, one of the observations was that centres with higher workload performed better. There is also existing evidence in Cancer literature that centres with higher surgical caseload have better outcomes [24]. There has also been a recent evaluation of the impact on stroke outcome by evidence-based practice in an Australian setting [25]. Examples of service delivery that are associated with better outcomes include organised stroke unit care [26], thrombolysis treatment and appropriate secondary prevention [27], and early supported discharge

in selected patients [28,29]. However, the cost-effectiveness of such services has yet to be fully examined.

Rodgers et al [30] highlighted the need for improvement in hospital-based stroke services e.g. stroke unit staffing levels were lower than was available in RCTs. The accumulating body of evidence has been a major driving force behind the UK Government's strategy to improve stroke care (National Stroke Strategy, 2007) [31]. A key strand of the strategy was to set up stroke networks to deliver stroke service development across geographically defined areas. The stroke networks have worked to agree minimum standards for stroke care and they have worked with commissioners to assist the commissioning process for stroke services. The acute stroke services are currently delivered by different NHS trusts and there is therefore a wide range of inequality in service availability and provision with differeing structure and local support systems.

This research aims to utilise NHS data in the most meaningful and innovative way and we aim to maximize the benefit with minimum investment to produce best research output for patient care by collaborating with clinical teams and the network in providing excellent value for money. This observational study seeks to identify areas of clinical practice which merit future randomised controlled trials (RCTs) to identify best practice in improving stroke care which will be of maximum benefit to patients. We also aim to obtain preliminary data to estimate sample sizes and conduct value of information analyses to design future pragmatic RCTs of innovative ways of delivering stroke care.

As we include eight diverse NHS trusts, the findings are likely to be generalisable in the UK setting and beyond. This study will provide observational data about health service factors associated with variations in patient outcomes and health care costs following hospital admission for acute stroke. This will form the basis for future RCTs by identifying promising health service interventions, assessing the feasibility of recruiting and following up trial patients, and provide evidence about frequency and variances in outcomes, and intra-cluster correlation of outcomes, for sample size calculations. The results will also inform clinicians, public, service providers, commissioners and policy makers to drive further improvement in health services and bring direct benefit to patients.

The study will describe the variation in outcomes between different stroke services, and identify the characteristics of services associated with better outcomes after accounting for case-mix. We will also estimate the relative costs of and health gain estimated as Quality Adjusted Life Year (QALY) gain that may be demonstrated by different services. The commissioners of services will be informed as to which service delivery structures are likely to provide value for money to make purchasing decisions. They will also be better informed about the types of service associated with better patient reported outcome. Hospital trusts will be able to evaluate their services systematically and plan their care appropriately to meet local and regional needs and demands based on our study findings. Professionals will be able to reflect on the impact of services they are delivering to help improve their performance and the way services are organised by adopting the most effective and cost effective approaches. As an observational study, the study limitations include inability to control for unknown confounders and residual confounding effect of known confounders which are adjusted for. The causal relationship cannot be implied but as we stated the findings will provide knowledge about areas that requires further evaluation in clinical trial setting.

There is very little work which assesses service provision robustly against patients' own reported outcomes. This exciting study may lead to a clearer drive for patients to define what makes a good service. We hope that the best clinical practices are adopted to suit the local populations' needs and demand. As we included eight diverse NHS trusts, the findings will be generalisable in the UK setting and likely to be applicable in international setting. All these will become drivers of improvement in stroke services for the benefit of stroke sufferers.

Acknowledgements
We would like to thank the participants of the study. We gratefully acknowledge the contribution of Stroke Research Nurses and the study steering committee members including representatives from the Regional Stroke Association, and Patient and Public Involvement in Research Panel. We would like to thank our colleagues from all participating trusts, site data co-ordinator and staff from Anglia Stroke & Heart Network for their assistance. Norfolk and Norwich University Hospital NHS Foundation Trust sponsors the study.

Manuscripts that are under submission based on this protocol
None.

Author details
[1]Norwich Medical School, Faculty of Medicine & Health Sciences, Norwich, UK. [2]Norfolk & Norwich University Hospital, Norwich, UK. [3]Anglia Stroke & Heart Clinical Network, Cambridge, UK. [4]James Paget University Hospital, Lowestoft, UK. [5]Queen Elizabeth Hospital, King's Lynn, UK. [6]Peterborough City Hospital, Peterborough, UK. [7]Hinchingbrooke Hospital, Huntingdon, UK. [8]Ipswich Hospital, Ipswich, UK. [9]West Suffolk Hospital, Bury St Edmund, UK. [10]Addenbrooke's University Hospital, Cambridge, UK.

Authors' contributions
PKM, DJD, MOB designed the outline of the study. PKM, JFP, MOB, EAW, GMP, GAB and AKM obtained the funding for the study. SDM & RH contributed in protocol preparation. All authors contributed in writing of the paper. All authors read and approved the final manuscript. PKM is the guarantor.

Competing interests
The authors declare that they have no competing interests.

Received: 21 January 2011 Accepted: 28 February 2011
Published: 28 February 2011

References

1. National Institute for Health and Clinical Excellence: **STROKE: National clinical guideline for diagnosis and initial management of acute stroke and transient ischaemic attack (TIA). The National Collaborative Centre for Chronic Conditions.** *Royal College of Physicians of London* 2008 [http://www.nice.org.uk/nicemedia/pdf/CG68FullGuideline.pdf], ISBN 978-1-86016-339-5.
2. National Audit Office: **Reducing brain damage: faster access to better stroke care.** *London: Stationery Office, 2005* National Audit Office Report; 2005.
3. Rothwell PM, Coull AJ, Silver LE, Fairhead JF, Giles MF, Lovelock CE, Redgrave JN, Bull LM, Welch SJ, Cuthbertson FC, Binney LE, Gutnikov SA, Anslow P, Banning AP, Mant D, Mehta Z, Oxford Vascular Study: **Population-based study of event-rate, incidence, case fatality and mortality for all acute vascular events in all arterial territories (Oxford Vascular Study).** *Lancet* 2005, **366**:1773-83.
4. Grieve R, Hutton J, Bhalla A, Rastenytë D, Ryglewicz D, Sarti C, Lamassa M, Giroud M, Dundas R, Wolfe CD: **A comparison of the costs and survival of hospital-admitted stroke patients across Europe.** *Stroke* 2001, **32**:1684-91.
5. Weir NU, Sandercock PA, Lewis SC, Signorini DF, Warlow CP: **Variations between countries in outcome after stroke in the International Stroke Trial (IST).** *Stroke* 2001, **32**:1370-7.
6. Gray LJ, Sprigg N, Bath PM, Sørensen P, Lindenstrøm E, Boysen G, De Deyn PP, Friis P, Leys D, Marttila R, Olsson JE, O'Neill D, Ringelstein B, van der Sande JJ, Turpie AG, TAIST Investigators: **Significant variation in mortality and functional outcome after acute ischaemic stroke between Western countries: data from the tinzaparin in acute ischaemic stroke trial (TAIST).** *J Neurol Neurosurg Psychiatry* 2006, **77**:327-33.
7. Markus H: **Improving the outcome of stroke.** *BMJ* 2007, **335**:359-60.
8. Rudd AG, Irwin P, Rutledge Z, Lowe D, Wade DT, Pearson M: **Regional variations in stroke care in England, Wales and Northern Ireland: results from the National Sentinel Audit of Stroke. Royal College of Physicians Intercollegiate Stroke Working Party.** *Clin Rehabil* 2001, **15**:562-72.
9. Rudd AG, Lowe D, Hoffman A, Irwin P, Pearson M: **Secondary prevention for stroke in the United Kingdom: results from the National Sentinel Audit of Stroke.** *Age Ageing* 2004, **33**:280-6.
10. Rudd AG, Hoffman A, Irwin P, Pearson M, Lowe D, Intercollegiate Working Party for Stroke: **Stroke units: research and reality. Results from the National Sentinel Audit of Stroke.** *Qual Saf Health Care* 2005, **14**:7-12.
11. Howell E, Graham C, Hoffman A, Lowe D, McKevitt C, Reeves R, Rudd AG: **Comparison of patients' assessments of the quality of stroke care with audit findings.** *Qual Saf Health Care* 2007, **16**:450-5.
12. National Sentinel Stroke Audit: **Phase II (clinical audit), 2008. Report for England, Wales and Northern Ireland. Intercollegiate Stroke Working Party.** *Clinical Effectiveness and Evaluation Unit, Royal College of Physicians of London* 2009.
13. Myint PK, Vowler SL, Woodhouse PR, Redmayne O, Fulcher RA: **Winter excess in hospital admissions, in-patient mortality and length of acute hospital stay in stroke: a hospital database study over six seasonal years in Norfolk, UK.** *Neuroepidemiology* 2007, **28**:79-85.
14. Myint PK, Kamath AV, Vowler SL, Maisey DN, Harrison BD: **The CURB (confusion, urea, respiratory rate and blood pressure) criteria in community-acquired pneumonia (CAP) in hospitalised elderly patients aged 65 years and over: a prospective observational cohort study.** *Age Ageing* 2005, **34**:75-7.
15. Myint PK, Vowler SL, Redmayne O, Fulcher RA: **Utilisation of diagnostic computerised tomography imaging and immediate clinical outcomes in older people with stroke before and after introduction of the National Service Framework for older people. A comparative study of hospital-based stroke registry data (1997-2003): Norfolk experience.** *Age Ageing* 2006, **35**:399-403.
16. Myint PK, Vowler SL, Redmayne O, Fulcher RA: **Cognition, continence and transfer status at the time of discharge from an acute hospital setting and their associations with an unfavourable discharge outcome after stroke.** *Gerontology* 2008, **54**:202-9.
17. Brooks R: **EuroQol: the current state of play.** *Health Policy* 1996, **37**:53-72.
18. National Institute for Clinical Excellence: 2004 [http://www.nice.org.uk/aboutnice/howwework/devnicetech/technologyappraisalprocessguides/guide_to_the_methods_of_technology_appraisal_reference_n0515.jsp], (reference N0515). ISBN: 1-84257-595-3.
19. Barton GR, Sach TH, Jenkinson C, Avery AJ, Doherty M, Muir KR: **Do estimates of cost-utility based on the EQ-5 D differ from those based on the mapping of utility scores?** *Health Qual Life Outcomes* 2008, **6**:51.
20. Barton GR, Briggs AH, Fenwick EA: **Optimal cost-effective decisions: the role of the cost-effectiveness acceptability curve (CEAC), cost-effectiveness acceptability frontier (CEAF) and expected value of perfect information (EVPI).** *Value Health* 2008, **11**:886-897.
21. Wardlaw JM, Seymour J, Cairns J, Keir S, Lewis S, Sandercock P: **Immediate computed tomography scanning of acute stroke is cost-effective and improves quality of life.** *Stroke* 2004, **35**:2477-83.
22. Audebert HJ, Kukla C, Vatankhah B, Gotzler B, Schenkel J, Hofer S, Fürst A, Haberl RL: **Comparison of tissue plasminogen activator administration management between Telestroke Network hospitals and academic stroke centers: the Telemedical Pilot Project for Integrative Stroke Care in Bavaria/Germany.** *Stroke* 2006, **37**:1822-7.
23. Lees KR, Ford GA, Muir KW, Ahmed N, Dyker AG, Atula S, Kalra L, Warburton EA, Baron JC, Jenkinson DF, Wahlgren NG, Walters MR, SITS-UK Group: **Thrombolytic therapy for acute stroke in the United Kingdom: experience from the safe implementation of thrombolysis in stroke (SITS) register.** *QJM* 2008, **101**:863-9.
24. Bachmann MO, Alderson D, Edwards D, Wotton S, Bedford C, Peters TJ, Harvey IM: **Cohort study in South and West England of the influence of specialization on the management and outcome of patients with oesophageal and gastric cancers.** *Br J Surg* 2002, **89**:914-22.
25. Gattellari M, Worthington J, Jalaludin B, Mohsin M: **Stroke unit care in a real-life setting: can results from randomized controlled trials be translated into every-day clinical practice? An observational study of hospital data in a large Australian population.** *Stroke* 2009, **40**:10-7.
26. Stroke Unit Trialists' Collaboration: **Organised inpatient (stroke unit) care for stroke.** *Cochrane Database Syst Rev* 2007, , **4**: CD000197, Review.
27. Higgins P, Lees KR: **Advances in emerging therapies.** *Stroke* 2009, **40(5)**: e292-4.
28. Langhorne P, Taylor G, Murray G, Dennis M, Anderson C, Bautz-Holter E, Dey P, Indredavik B, Mayo N, Power M, Rodgers H, Ronning OM, Rudd A, Suwanwela N, Widen-Holmqvist L, Wolfe C: **Early supported discharge services for stroke patients: a meta-analysis of individual patients' data.** *Lancet* 2005, **365**:501-6.
29. Early Supported Discharge Trialists: **Services for reducing duration of hospital care for acute stroke patients.** *Cochrane Database Syst Rev* 2005, , **2**: CD000443.
30. Rodgers H, Dennis M, Cohen D, Rudd A, British Association of Stroke Physicians: **British Association of Stroke Physicians: benchmarking survey of stroke services.** *Age Ageing* 2003, **32**:211-7.
31. National Stroke Strategy: **Department of Health.** 2007 [http://www.dh.gov.uk/en/Publicationsandstatistics/Publications/PublicationsPolicyandGuidance/DH_081062].

Pre-publication history

The pre-publication history for this paper can be accessed here:
http://www.biomedcentral.com/1472-6963/11/50/prepub

doi:10.1186/1472-6963-11-50
Cite this article as: Myint et al.: Evaluation of stroke services in Anglia stroke clinical network to examine the variation in acute services and stroke outcomes. *BMC Health Services Research* 2011 **11**:50.

Submit your next manuscript to BioMed Central and take full advantage of:

- **Convenient online submission**
- **Thorough peer review**
- **No space constraints or color figure charges**
- **Immediate publication on acceptance**
- **Inclusion in PubMed, CAS, Scopus and Google Scholar**
- **Research which is freely available for redistribution**

Submit your manuscript at
www.biomedcentral.com/submit

BioMed Central
