# Supplementary material for: Does service heterogeneity have an impact on acute hospital length of stay in stroke? A UK-based multicentre prospective cohort study
Source: BMJ Open. 2019 Apr 3;9(4):e024506. doi: 10.1136/bmjopen-2018-024506 (PMC6500188; doi:10.1136/bmjopen-2018-024506)
Supplement: Supplementary data [file bmjopen-2018-024506supp002.pdf]

Supplementary document to:

Does service heterogeneity have an impact on acute hospital length of stay in stroke? A UK-based multi-centre prospective cohort study

Michelle Tørnes, David McLernon, Max O Bachmann, Stanley D Musgrave, Elizabeth A Warburton, John F Potter, Phyo Kyaw Myint: On behalf of the Anglia Stroke Clinical Network Evaluation Study (ASCNES) Group

CONTENT

Table S1 Variables used to inform multiple imputation of missing data

Table S2 Sample characteristics distribution of the 2333 patients included in analysis per individual hospital (n (%) unless otherwise stated)

Table S3 Sample characteristics of complete cases and those with at least one variable missing

Table S4 Univariable regression analysis for multiple imputed dataset for AHLOS (n=2233)

Table S5 Univariable linear regression complete case analysis for AHLOS

Table S6 Multiple linear regression complete case analysis for AHLOS (n=1496, R^2=44.7%)

Table S7 Multiple linear regression sensitivity analysis for AHLOS, excluding Hospital 2 using multiple imputed dataset (n=2217, R^2=44.7%)

Table S8 Multiple linear regression sensitivity analysis for AHLOS, including discharge destination using multiple imputed dataset (n=2233, R^2=40%)

Figure S1 Model estimates of mean baseline acute hospital length of stay (AHLOS) per hospital (in days) and number of stroke patients treated outside the stroke unit per day per five stroke unit beds with 95% confidence intervals. Multiple regression model was adjusted for patient covariates that had a p-value<0.3 in univariable analysis.

Figure S2 Model estimates of mean baseline acute hospital length of stay (AHLOS) per hospital (in days) and presence of vascular surgery onsite with 95% confidence intervals. Multiple regression model was adjusted for patient covariates that had a p-value<0.3 in univariable analysis.

Figure S3 Model estimates of mean baseline acute hospital length of stay (AHLOS) per hospital (in days) and distance to neurosurgical facility with 95% confidence intervals. Multiple regression model was adjusted for patient covariates that had a p-value<0.3 in univariable analysis.

Figure S4 Model estimates of mean baseline acute hospital length of stay (AHLOS) per hospital (in days) and number of fte senior doctors per five beds available during weekdays with 95% confidence intervals. Multiple regression model was adjusted for patient covariates that had a p-value<0.3 in univariable analysis.

Figure S5 Model estimates of mean baseline acute hospital length of stay (AHLOS) per hospital (in days) and number of fte junior doctors per five beds available during weekdays with 95% confidence intervals. Multiple regression model was adjusted for patient covariates that had a p-value<0.3 in univariable analysis.

Figure S6 Model estimates of mean baseline acute hospital length of stay (AHLOS) per hospital (in days) and number of fte health care associates and nurses per five beds with 95% confidence intervals. Multiple regression model was adjusted for patient covariates that had a p-value<0.3 in univariable analysis.

Figure S7 Model estimates of mean baseline acute hospital length of stay (AHLOS) per hospital (in days) and number of fte occupational therapists per five beds with 95% confidence intervals. Multiple regression model was adjusted for patient covariates that had a p-value<0.3 in univariable analysis.

Figure S8 Model estimates of mean baseline acute hospital length of stay (AHLOS) per hospital (in days) and number of fte physiotherapists per five beds with 95% confidence intervals. Multiple regression model was adjusted for patient covariates that had a p-value<0.3 in univariable analysis.

Figure S9 Model estimates of mean baseline acute hospital length of stay (AHLOS) per hospital (in days) and number of fte speech and language therapists per five beds with 95% confidence intervals. Multiple regression model was adjusted for patient covariates that had a p-value<0.3 in univariable analysis.

Figure S10 Model estimates of mean baseline acute hospital length of stay (AHLOS) per hospital (in days) and number of total beds present on stroke unit per 100 admissions with 95% confidence intervals. Multiple regression model was adjusted for patient covariates that had a p-value<0.3 in univariable analysis.

Figure S11 Model estimates of mean baseline acute hospital length of stay (AHLOS) per hospital (in days) and number of hospital beds per computed tomography (CT) scanner with 95% confidence intervals. Multiple regression model was adjusted for patient covariates that had a p-value<0.3 in univariable analysis.

Figure S12 Model estimates of mean baseline acute hospital length of stay (AHLOS) per hospital (in days) and provision of onsite rehabilitation service with 95% confidence intervals. Multiple regression model was adjusted for patient covariates that had a p-value<0.3 in univariable analysis.

Figure S13 Model estimates of mean baseline acute hospital length of stay (AHLOS) per hospital (in days) and presence of early supported discharge scheme with 95% confidence intervals. Multiple regression model was adjusted for patient covariates that had a p-value<0.3 in univariable analysis.

Figure S14 Model estimates of mean baseline acute hospital length of stay (AHLOS) per hospital (in days) and number of non-stroke patients present on the stroke unit per day per five stroke unit beds with 95% confidence intervals. Multiple regression model was adjusted for patient covariates that had a p-value<0.3 in univariable analysis.

Figure S15 Model estimates of mean baseline acute hospital length of stay (AHLOS) per hospital (in days) and mean Index of Multiple Deprivation (IMD) score of the counties in which the hospital serves with 95% confidence intervals. Multiple regression model was adjusted for patient covariates that had a p-value<0.3 in univariable analysis.

Table S1 Variables used to inform multiple imputation of missing data

Variable	Measure
I. Independent Variables	
Trust	0=Trust 1 1 =Trust 2 2 =Trust 3 3=Trust 4 4=Trust 5 4=Trust 6 5=Trust 7 6=Trust 8
Sex	0=Male 1=Female
Age	Continuous, years
Recurrent Stroke	0=No 1=Yes
Diabetes Mellitus	0=No 1=Yes
Dementia	0=No 1=Yes
Hypercholesterolemia	0=No 1=Yes
Myocardial Infarction or Ischaemic Heart Disease	0=No 1=Yes
Transient Ischaemic Attack	0=No 1=Yes
Previous Cancer	0=No 1=Yes
Active Cancer	0=No 1=Yes
Depression	0=No 1=Yes
Rheumatoid Arthritis	0=No 1=Yes
Chronic Obstructive Pulmonary Disease	0=No 1=Yes
Pre-Stroke modified Rankin Score (mRS)	0=0 1=1 2=2 3=3 4=4 & 5
Pre-Stroke Residence	0=Independent living without formal care<br>1=Independent living with formal care<br>2=Institutional care
Stroke Type	0=Ischaemic 1=Haemorrhagic
Oxfordshire Community Stroke Classification	0=LACS 1=PACS 2=POCS 3=TACS
Brain Lateralisation	0=Yes 1=No
Inpatient Complication	0=No 1=Yes
Discharge modified Rankin Score (mRS)	0=0 1=1 2=2 3=3 4=4 5=5 6=6
Season of Admission	0=Summer 1=Winter
Day of Admission	0=Weekday 1=Weekend
II. Dependent Variable	
Logarithmic acute hospital LOS	Continuous, days
III. Auxiliary Variables	
Discharge Destination	0=Independent living without formal care<br>1=Independent living with formal care<br>2=Institutional care<br>3=Interim or rehabilitation setting<br>4=Death
Atrial Fibrillation	0=No 1=Yes
Baseline Systolic Blood Pressure	Continuous, mmHg
Baseline Diastolic Blood Pressure	Continuous, mmHg
Glucose Concentration on Admission	Continuous, mmol/L
Weight	Continuous, kg
Heart Rate	Continuous, beats per minute
Temperature	Continuous, ˚C
Oxygen Saturation	Continuous, %
ITU or HDU admission	0. No 1. Yes
Systolic Blood Pressure at Discharge	Continuous, mmHg
Diastolic Blood Pressure at Discharge	Continuous, mm Hg

LOS, Length of Stay; ITU, Intensive Care Unit; HDU, High Dependency Unit.

Table S2 Sample characteristics of the 2333 patients included in analysis per individual hospital (n (%) unless otherwise stated)

Variables	Hospital 1 350 (16)	Hospital 2 16 (1)	Hospital 3 350 (16)	Hospital 4 143 (6)	Hospital 5 618 (28)	Hospital 6 281 (13)	Hospital 7 252 (11)	Hospital 8 223 (10)
Age, y, median (IQR)	78 (68 to 85)	87 (81 to 92)	79 (72 to 86)	79 (70 to 86)	79 (71 to 85)	78 (71 to 85)	80 (68 to 85)	80 (71 to 87)
Sex, female	180 (52)	9 (56)	197 (56)	76 (53)	309 (50)	155 (55)	116 (46)	123 (55)
Recurrent Stroke	50 (14)	5 (31)	61 (17)	19 (17)	143 (23)	62 (22)	66 (26)	42 (19)
Diabetes Mellitus	48 (14)	1 (6)	59 (17)	17 (15)	92 (15)	66 (23)	44 (17)	43 (19)
Dementia	26 (7)	1 (6)	35 (10)	10 (9)	58 (9)	29 (10)	23 (9)	25 (11)
Hypercholesterolemia	48 (14)	3 (19)	24 (7)	7 (6)	61 (10)	80 (28)	38 (15)	94 (42)
Hypertensive	225 (64)	8 (50)	202 (58)	56 (50)	446 (72)	200 (71)	187 (74)	159 (71)
Myocardial Infarction or Ischaemic Heart Disease	45 (13)	3 (19)	87 (25)	30 (27)	142 (23)	80 (28)	49 (19)	81 (36)
Transient Ischaemic Attack	32 (9)	3 (19)	58 (17)	17 (15)	113 (18)	40 (14)	47 (19)	30 (13)
Previous Cancer	33 (9)	1 (6)	38 (11)	12 (11)	41 (7)	18 (6)	21 (8)	31 (14)
Active Cancer	24 (7)	2 (12)	8 (2)	10 (9)	49 (8)	9 (3)	20 (8)	15 (7)
Depression	13 (4)	0 (0)	17 (5)	8 (7)	33 (5)	11 (4)	18 (7)	17 (8)
Rheumatoid Arthritis	11 (3)	1 (6)	43 (12)	3 (3)	83 (13)	2 (1)	7 (3)	4 (2)
COPD	15 (4)	1 (6)	20 (6)	6 (5)	26 (4)	20 (7)	11 (4)	17 (8)
Pre-stroke mRS Score								
0	84 (43)	3 (19)	117 (36)	-	330 (56)	126 (64)	136 (56)	118 (53)
1	60(31)	3 (19)	75 (23)	-	87 (15)	16 (8)	61 (25)	33 (15)
2	24 (12)	3 (19)	51 (16)	-	56 (9)	17 (9)	16 (7)	24 (11)
3	21 (11)	2 (12)	38 (12)	-	60 (10)	20 (10)	15 (6)	28 (13)
4 & 5	7 (4)	5 (31)	44 (14)	-	57 (10)	18 (9)	16 (7)	20 (9)
Pre-Stroke Residence								
Independent living with formal care	21 (6)	4 (25)	23 (7)	15 (14)	62 (10)	30 (11)	34 (13)	21 (10)
Independent living w/o formal care	292 (86)	9 (56)	285 (82)	86 (77)	493 (80)	215 (77)	193 (77)	179 (82)
Institution	28 (8)	3 (19)	40 (11)	10 (9)	63 (10)	35 (12)	23 (9)	18 (8)

Variables	Hospital 1<br>350<br>(16)	Hospital 2<br>16<br>(1)	Hospital 3<br>350<br>(16)	Hospital 4<br>143<br>(6)	Hospital 5<br>618<br>(28)	Hospital 6<br>281<br>(13)	Hospital 7<br>252<br>(11)	Hospital 8<br>223<br>(10)
Stroke Type								
Ischaemic	293 (85)	14 (100)	286 (87)	90 (91)	541 (88)	233 (85)	213 (87)	194 (88)
Haemorrhagic	50 (15)	0 (0)	43 (13)	9 (9)	73 (12)	40 (15)	32 (13)	26(12)
Oxford Community Stroke Project Classification								
LACS	64 (24)	1 (7)	95 (29)	20 (28)	149 (25)	51 (18)	39 (19)	84 (39)
PACS	117 (43)	11 (79)	109 (33)	38 (54)	216 (37)	147 (53)	80 (39)	66 (30)
POCS	51 (19)	-	29 (9)	3 (4)	117 (20)	21 (8)	33 (16)	25 (12)
TACS	38 (14)	2 (14)	99 (30)	10 (14)	107 (18)	57 (21)	52 (25)	42 (19)
No Brain Lateralisation	50 (15)	2 (13)	14 (4)	9 (9)	129 (21)	1 (0.4)	30 (12)	9 (4)
Inpatient Complication	108 (31)	4 (25)	34 (10)	36 (25)	229 (37)	109 (39)	83 (33)	52 (23)
Discharge mRS Score								
0	37 (15)	0 (0)	11 (3)	0 (0)	114 (19)	34 (16)	42 (17)	22 (10)
1	65 (25)	2 (12)	55 (17)	0 (0)	97 (16)	25 (12)	55 (23)	53 (24)
2	36 (14)	1 (6)	46 (14)	0 (0)	57 (10)	20 (9)	33 (14)	19 (9)
3	41 (16)	4 (25)	40 (12)	0 (0)	87 (15)	36 (17)	34 (14)	49 (22)
4	19 (7)	3 (19)	57 (17)	0 (0)	89 (15)	25 (12)	16 (7)	29 (13)
5	4 (2)	1 (6)	47 (14)	0 (0)	40 (7)	14 (7)	16 (7)	15 (7)
6	53 (21)	5 (31)	77 (23)	29 (100)	110 (19)	58 (27)	47 (19)	35 (16)
Winter Admission	172 (49)	16 (100)	181 (52)	73 (51)	332 (54)	140 (50)	131 (52)	114 (51)
Weekend Admission	113 (32)	3 (19)	98 (28)	43 (30)	177 (29)	74 (26)	55 (22)	51 (23)

IQR, Interquartile Range; COPD, Chronic Obstructive Pulmonary Disease; mRS, modified Rankin Scale; LACS, Lacunar Anterior Circulation Stroke; PACS, Partial Anterior Circulation Stroke; POCS, Posterior Circulation Stroke; TACS, Total Anterior Circulation Stroke.

Table S3 Sample characteristics of complete cases and those with at least one variable missing

Patient Characteristic	Complete Cases (n=1496)	Cases with at least one missing variable	*P*
	Median (IQR) or No. (%)		
Age, y*	79 (71 to 86)	79 (70 to 86)	0.34
Sex, female†	781 (52)	384 (52)	1
Comorbidities†			
Recurrent Stroke	328 (22)	120 (17)	0.01
Diabetes Mellitus	259 (17)	111 (16)	0.38
Dementia	138 (9)	69 (10)	0.75
Hypercholesterolemia	264 (18)	91 (13)	0.01
Hypertensive	1054 (70)	429 (61)	<0.001
Myocardial Infarction or Ischaemic Heart Disease	362(24)	155 (22)	0.26
TIA	248 (17)	92 (13)	0.04
Previous Cancer	140 (9)	55 (8)	0.25
Active Cancer	93 (6)	44 (6)	1
Depression	79 (5)	38 (5)	1
Rheumatoid Arthritis	129 (9)	25 (3)	<0.001
COPD	76 (5)	40 (6)	0.64
Pre-stroke mRS Score‡			0.62
0	765 (51)	149 (51)	
1	284 (19)	51 (17)	
2	167 (11)	24 (8)	
3	149 (10)	35 (12)	
4 & 5	131 (9)	36 (12)	
Pre-stroke Residence†			<0.001
Independent living with formal care	145 (10)	65 (9)	
Independent living without formal	1215 (81)	537 (78)	
Institution	136 (9)	84 (12)	
Haemorrhagic Stroke†	138 (9)	135 (21)	<0.001
Oxford Community Stroke Project‡			0.05
LACS	411 (27)	92 (19)	
PACS	570 (38)	214 (45)	
POCS	214 (14)	65 (14)	
TACS	301 (20)	106 (22)	
No Brain Lateralisation†	174 (12)	70 (12)	0.74
Inpatient Complication†	421 (28)	234 (32)	0.09
Discharge mRS Score‡			0.02
0	218 (15)	42 (10)	
1	295 (20)	57 (14)	
2	177 (12)	35 (9)	
3	243 (16)	48 (12)	
4	209 (14)	29 (7)	
5	121 (8)	16 (4)	
6	233 (16)	181 (44)	

Winter Admission†	770 (51)	389 (53)	0.59
Weekend Admission†	401 (27)	213 (29)	0.32

IQR, Interquartile Range; TIA, Transient Ischaemic Attack; COPD, Chronic Obstructive Pulmonary Disease; mRS, modified Rankin Scale; LACS, Lacunar Anterior Circulation Stroke; PACS, Partial Anterior Circulation Stroke; POCS, Posterior Circulation Stroke; TACS, Total Anterior Circulation Stroke.

**Two sample t-test*

† $\mathcal{X}^2$ test

‡ $\mathcal{X}^2$ test for trend

Table S4 Univariable regression analysis for multiple imputed dataset for AHLOS (n=2233)

Patient Characteristic	β	95% CI	*P*	R^2
Age, y	1.02	1.02 to 1.02	<0.001	4.8
Sex, female	1.20	1.10 to 1.31	<0.001	0.7
Recurrent Stroke	1.17	1.05 to 1.31	0.01	0.4
Diabetes Mellitus	1.16	1.03 to 1.31	0.02	0.3
Dementia	1.46	1.25 to 1.70	<0.001	1.1
Hypercholesterolemia	0.84	0.75 to 0.95	0.01	0.3
Hypertensive	1.02	0.93 to 1.12	0.66	0
Myocardial Infarction/ Ischaemic Heart Disease*	1.07	0.96 to 1.19	0.23	0.1
TIA	1.07	0.94 to 1.21	0.30	0.1
Previous Cancer	1.23	1.05 to 1.44	0.01	0.3
Active Cancer	0.97	0.80 to 1.16	0.72	0
Depression	1.06	0.86 to 1.29	0.59	0
Rheumatoid Arthritis	1.10	0.92 to 1.31	0.31	0.1
COPD	0.86	0.71 to 1.06	0.15	0.1
Pre-stroke mRS Score (reference 0)			<0.001	5.5
1	1.57	1.38 to 1.79	<0.001	
2	1.63	1.39 to 1.91	<0.001	
3	1.94	1.65 to 2.28	<0.001	
4 & 5	1.32	1.13 to 1.55	<0.001	
Pre-stroke Residence (reference Independent living w/o formal care)			<0.001	1.4
Independent living with formal care	1.52	1.31 to 1.77	<0.001	
Institution	1.13	0.97 to 1.31	0.11	
Haemorrhagic Stroke	0.83	0.73 to 0.96	0.01	0.3
Oxford Community Stroke Project Classification (reference LACS)			<0.001	4.0
PACS	1.62	1.44 to 1.82	<0.001	
POCS	1.22	1.05 to 1.42	0.01	
TACS	1.66	1.45 to 1.90	<0.001	
Brain Lateralisation	0.69	0.60 to 0.80	<0.001	1.2
Inpatient Complication	2.13	1.94 to 2.34	<0.001	10.3
Discharge mRS Score (reference 0)			<0.001	31.1
1	1.24	1.07 to 1.42	0.003	
2	2.04	1.75 to 2.39	<0.001	
3	3.35	2.90 to 3.87	<0.001	
4	4.20	3.60 to 4.90	<0.001	
5	6.67	5.62 to 7.91	<0.001	
6	1.57	1.37 to 1.80	<0.001	
Winter Admission	1.20	1.09 to 1.31	<0.001	0.7
Weekend Admission	1.08	0.98 to 1.20	0.12	0.1
Hospital (reference 1)			<0.001	2.4
2	2.69	1.58 to 4.58	<0.001	
3	1.19	1.02 to 1.39	0.03	
4	1.24	1.01 to 1.53	0.04	
5	0.86	0.75 to 0.99	0.03	
6	1.11	0.94 to 1.31	0.22	
7	1.18	1.00 to 1.41	0.05	

Patient Characteristic	β	95% CI	*P*	R^2
8	0.86	0.72 to 1.03	0.11	

AHLOS, Acute Hospital Length of Stay; CI, Confidence Interval; TIA, Transient Ischaemic Attack; COPD, Chronic Obstructive Pulmonary Disease; mRS, modified Rankin Scale; LACS, Lacunar Anterior Circulation Stroke; PACS, Partial Anterior Circulation Stroke; POCS, Posterior Circulation Stroke; TACS, Total Anterior Circulation Stroke.

Table S5 Univariable linear regression complete case analysis for AHLOS

Patient Characteristic	N	β	95% CI	*P*	% R^2
Age, y	2231	1.02	1.02 to 1.02	<0.001	4.7
Sex, female	1165 v. 1066	1.20	1.10 to 1.31	<0.001	0.7
Recurrent Stroke	448 v. 1755	1.17	1.05 to 1.31	0.005	0.3
Diabetes Mellitus	370 v. 1833	1.16	1.03 to 1.31	0.02	0.2
Dementia	207 v. 1996	1.46	1.25 to 1.70	<0.001	1.0
Hypercholesterolemia	355 v. 1848	0.85	0.75 to 0.95	0.01	0.3
Hypertensive	1483 v. 720	1.03	0.93 to 1.13	0.57	0
Myocardial Infarction or Ischaemic Heart Disease*	517 v. 1686	1.07	0.96 to 1.19	0.23	0
TIA	340 v. 1863	1.06	0.94 to 1.20	0.32	0
Previous Cancer	195 v. 2008	1.23	1.05 to 1.44	0.01	0.3
Active Cancer	137 v. 2066	0.96	0.80 to 1.15	0.65	0
Depression	117 v. 2086	1.05	0.86 to 1.28	0.65	0
Rheumatoid Arthritis	154 v. 2049	1.10	0.92 to 1.31	0.31	0
COPD	116 v. 2087	0.86	0.70 to 1.05	0.14	0.1
Pre-stroke mRS Score (reference 0)				<0.001	5.8
1	335 v. 914	1.58	1.39 to 1.80	<0.001	
2	191 v. 914	1.62	1.38 to 1.90	<0.001	
3	184 v. 914	1.97	1.67 to 2.31	<0.001	
4 & 5	167 v. 914	1.45	1.22 to 1.71	<0.001	
Pre-stroke Residence (reference Independent living without formal care)				<0.001	1.3
Independent living with formal care	210 v. 1752	1.52	1.31 to 1.77	<0.001	
Institution	220 v. 1752	1.14	0.98 to 1.32	0.09	
Haemorrhagic Stroke	273 v. 1864	0.85	0.74 to 0.97	0.02	0.2
Oxford Community Stroke Project Classification (reference LACS)				<0.001	4.3
PACS	784 v. 503	1.62	1.44 to 1.82	<0.001	
POCS	279 v. 503	1.24	1.06 to 1.44	0.01	
TACS	407 v. 503	1.75	1.53 to 2.01	<0.001	
No Brain Lateralisation	244 v. 1822	0.68	0.59 to 0.79	<0.001	1.3
Inpatient Complication	655 v. 1578	2.13	1.94 to 2.34	<0.001	10
Discharge mRS Score (reference 0)				<0.001	30.1
1	352 v. 260	1.25	1.08 to 1.44	0.002	
2	212 v. 260	2.01	1.72 to 2.36	<0.001	
3	291 v. 260	3.30	2.84 to 3.82	<0.001	
4	238 v. 260	4.17	3.57 to 4.87	<0.001	
5	137 v. 260	6.97	5.81 to 8.37	<0.001	
6	414 v. 260	1.58	1.38 to 1.81	<0.001	
Winter Admission	1159 v. 1074	1.20	1.09 to 1.31	<0.001	0.6
Weekend Admission	614 v. 1619	1.08	0.98 to 1.20	0.12	0.1
Hospital (reference 1)				<0.001	2.1
2	16 v. 350	2.69	1.58 to 4.58	<0.001	
3	350 v. 350	1.19	1.02 to 1.39	0.03	
4	143 v. 350	1.24	1.01 to 1.53	0.04	
5	618 v. 350	0.86	0.75 to 0.99	0.03	
6	281 v. 350	1.11	0.94 to 1.31	0.22	
7	252 v. 350	1.18	1.00 to 1.41	0.05	

Patient Characteristic	N	β	95% CI	*P*	% R^2
8	223 v. 350	0.86	0.72 to 1.03	0.11	

AHLOS, Acute Hospital Length of Stay; CI, Confidence Interval; TIA, Transient Ischaemic Attack; COPD, Chronic Obstructive Pulmonary Disease; mRS, modified Rankin Scale; LACS, Lacunar Anterior Circulation Stroke; PACS, Partial Anterior Circulation Stroke; POCS, Posterior Circulation Stroke; TACS, Total Anterior Circulation Stroke.

Table S6 Multiple *l*inear *r*egression *c*omplete *c*ase *a*nalysis for AHLOS (n=1496, R^2=44.7%).

Patient Characteristic	N	β	95% CI	*P*
Age, y	1496	1.01	1.00 to 1.01	<0.001
Sex, female	781 v. 715	0.98	0.90 to 1.07	0.66
Recurrent Stroke	328 v. 1168	1.06	0.96 to 1.17	0.27
Diabetes Mellitus	259 v. 1237	0.99	0.89 to 1.11	0.91
Dementia	138 v. 1358	1.32	1.13 to 1.53	<0.001
Hypercholesterolemia	264 v. 1232	0.92	0.82 to 1.02	0.13
Myocardial Infarction or Ischaemic Heart Disease*	362 v. 1134	1.00	0.91 to 1.10	0.97
Previous Cancer	140 v. 1356	1.16	1.01 to 1.33	0.03
COPD	76 v. 1420	0.91	0.76 to 1.09	0.31
Pre-stroke mRS Score (reference 0)				<0.001
1	284 v. 765	1.08	0.96 to 1.20	0.21
2	167 v. 765	0.93	0.80 to 1.08	0.33
3	149 v. 765	1.00	0.84 to 1.19	0.99
4 & 5	131 v. 765	0.77	0.63 to 0.93	0.01
Pre-Stroke Residence (reference Independent living without formal care)				<0.001
Independent living with formal care	145 v. 1215	1.02	0.88 to 1.19	0.78
Institution	136 v. 1215	0.83	0.69 to 0.98	0.03
Haemorrhagic Stroke	138 v. 1358	0.83	0.72 to 0.96	0.01
Oxford Community Stroke Project Classification				<0.001
PACS	570 v. 411	1.27	1.15 to 1.40	<0.001
POCS	214 v. 411	1.29	1.13 to 1.47	<0.001
TACS	301 v. 411	1.36	1.19 to 1.57	<0.001
No Brain Lateralisation	174 v. 1322	0.93	0.81 to 1.05	0.24
Inpatient Complication	421 v. 1075	1.67	1.51 to 1.84	<0.001
Discharge mRS Score (reference 0)				<0.001
1	295 v. 218	1.15	1.00 to 1.32	0.05
2	177 v. 218	1.60	1.36 to 1.88	<0.001
3	243 v. 218	2.45	2.10 to 2.87	<0.001
4	209 v. 218	3.39	2.86 to 4.02	<0.001
5	121 v. 218	4.78	3.89 to 5.88	<0.001
6	233 v. 218	1.34	1.11 to 1.61	0.002
Winter Admission	770 v. 726	1.16	1.07 to 1.25	<0.001
Weekend Admission	401 v. 1095	1.06	0.97 to 1.15	0.23
Hospital (reference1)				<0.001
2	14 v. 111	2.08	1.35 to 3.21	0.001
3	278 v. 111	1.20	1.01 to 1.44	0.04
4	-	-	-	-
5	558 v. 111	0.84	0.71 to 0.98	0.03
6	142 v. 111	1.03	0.85 to 1.26	0.75
7	191 v. 111	1.35	1.13 to 1.62	0.001
8	202 v. 111	0.94	0.78 to 1.13	0.49

AHLOS, Acute Hospital Length of Stay; CI, Confidence Interval; COPD, Chronic Obstructive Pulmonary Disease; mRS, modified Rankin Scale; LACS, Lacunar Anterior Circulation Stroke; PACS, Partial Anterior Circulation Stroke; POCS, Posterior Circulation Stroke; TACS, Total Anterior Circulation Stroke.

Table S7 Multiple linear regression sensitivity analysis for AHLOS, excluding Hospital 2 using multiple imputed dataset (n=2217, R^2=44.7%).

Patient Characteristic	e^{β}*	95% CI*	*P*
Age, y	1.01	1.00 to 1.01	<0.001
Sex, female	1.01	0.94 to 1.08	0.86
Recurrent Stroke	1.02	0.93 to 1.12	0.68
Diabetes Mellitus	1.07	0.97 to 1.17	0.19
Dementia	1.30	1.13 to 1.48	<0.001
Hypercholesterolemia	0.95	0.86 to 1.05	0.33
Myocardial Infarction or Ischaemic Heart Disease*	1.00	0.91 to 1.08	0.92
Previous Cancer	1.13	0.99 to 1.27	0.06
COPD	0.90	0.77 to 1.06	0.21
Pre-stroke mRS Score (reference 0)			<0.001
1	1.08	0.96 to 1.21	0.19
2	0.90	0.78 to 1.04	0.16
3	0.94	0.79 to 1.10	0.47
4 & 5	0.69	0.58 to 0.83	<0.001
Pre-Stroke Residence (reference Independent living without formal care)			<0.001
Independent living with formal care	1.01	0.88 to 1.16	0.91
Institution	0.81	0.69 to 0.95	0.01
Haemorrhagic Stroke	0.80	0.71 to 0.90	<0.001
Oxford Community Stroke Project Classification (reference LACS)			<0.001
PACS	1.30	1.18 to 1.43	<0.001
POCS	1.34	1.18 to 1.53	<0.001
TACS	1.29	1.13 to 1.47	<0.001
No Brain Lateralisation	0.85	0.75 to 0.95	0.01
Inpatient Complication	1.70	1.57 to 1.85	<0.001
Discharge mRS Score (reference 0)			<0.001
1	1.15	1.00 to 1.32	0.04
2	1.74	1.48 to 2.04	<0.001
3	2.72	2.34 to 3.16	<0.001
4	3.56	3.02 to 4.20	<0.001
5	5.12	4.22 to 6.22	<0.001
6	1.25	1.05 to 1.48	0.01
Winter Admission	1.15	1.08 to 1.24	<0.001
Weekend Admission	1.03	0.95 to 1.11	0.48
Hospital (reference 1)			<0.001
3	1.08	0.94 to 1.22	0.29
4	1.07	0.89 to 1.29	0.40
5	0.78	0.70 to 0.87	<0.001
6	0.93	0.81 to 1.07	0.33
7	1.15	1.00 to 1.32	0.05
8	0.82	0.70 to 0.95	0.01

AHLOS, Acute Hospital Length of Stay; CI, Confidence Interval; COPD, Chronic Obstructive Pulmonary Disease; mRS, modified Rankin Scale; LACS, Lacunar Anterior Circulation Stroke; PACS, Partial Anterior Circulation Stroke; POCS, Posterior Circulation Stroke; TACS, Total Anterior Circulation Stroke.

Table S8 Multiple linear regression sensitivity analysis for AHLOS, including discharge destination using multiple imputed dataset (n=2233, R^2=40%).

Patient Characteristic	e^{β}*	95% CI*	*P*
Age, y	1.01	1.00 to 1.01	<0.001
Sex, female	0.99	0.92 to 1.07	0.80
Recurrent Stroke	1.00	0.91 to 1.10	1.00
Diabetes Mellitus	1.08	0.98 to 1.19	0.12
Dementia	1.20	1.05 to 1.38	0.01
Hypercholesterolemia	0.94	0.85 to 1.04	0.25
Myocardial Infarction or Ischaemic Heart	1.01	0.93 to 1.10	0.83
Previous Cancer	1.17	1.03 to 1.33	0.01
COPD	0.91	0.77 to 1.07	0.23
Pre-stroke mRS Score (reference 0)			<0.001
1	1.15	1.03 to 1.28	0.02
2	1.15	1.00 to 1.33	0.05
3	1.33	1.13 to 1.56	<0.001
4 & 5	1.15	0.96 to 1.38	0.12
Pre-Stroke Residence (reference Independent living without formal care)			<0.001
Independent living with formal care	0.86	0.75 to 0.99	0.04
Institution	0.52	0.44 to 0.62	<0.001
Haemorrhagic Stroke	0.84	0.75 to 0.95	<0.001
Oxford Community Stroke Project Classification (reference LACS)			<0.001
PACS	1.34	1.22 to 1.48	<0.001
POCS	1.44	1.26 to 1.63	<0.001
TACS	1.49	1.31 to 1.70	<0.001
No Brain Lateralisation	0.82	0.73 to 0.93	<0.001
Inpatient Complication	1.72	1.58 to 1.87	<0.001
Discharge Destination (reference Independent living without formal care)			<0.001
Independent living with formal care	1.99	1.74 to 2.27	<0.001
Institution	3.58	3.09 to 4.15	<0.001
Interim/Rehab Setting	2.18	1.94 to 2.46	<0.001
Death	0.85	0.74 to 0.97	0.02
Winter Admission	1.15	1.07 to 1.24	<0.001
Weekend Admission	1.04	0.96 to 1.13	0.30
Hospital (reference 1)			<0.001
2	2.76	1.80 to 4.22	<0.001
3	1.24	1.09 to 1.42	<0.001
4	1.36	1.15 to 1.61	<0.001
5	0.85	0.75 to 0.95	0.01
6	1.06	0.92 to 1.22	0.42
7	1.19	1.03 to 1.37	0.02
8	0.99	0.85 to 1.14	0.84

AHLOS, Acute Hospital Length of Stay; CI, Confidence Interval; COPD, Chronic Obstructive Pulmonary Disease; mRS, modified Rankin Scale; LACS, Lacunar Anterior Circulation Stroke; PACS, Partial Anterior Circulation Stroke; POCS, Posterior Circulation Stroke; TACS, Total Anterior Circulation Stroke.

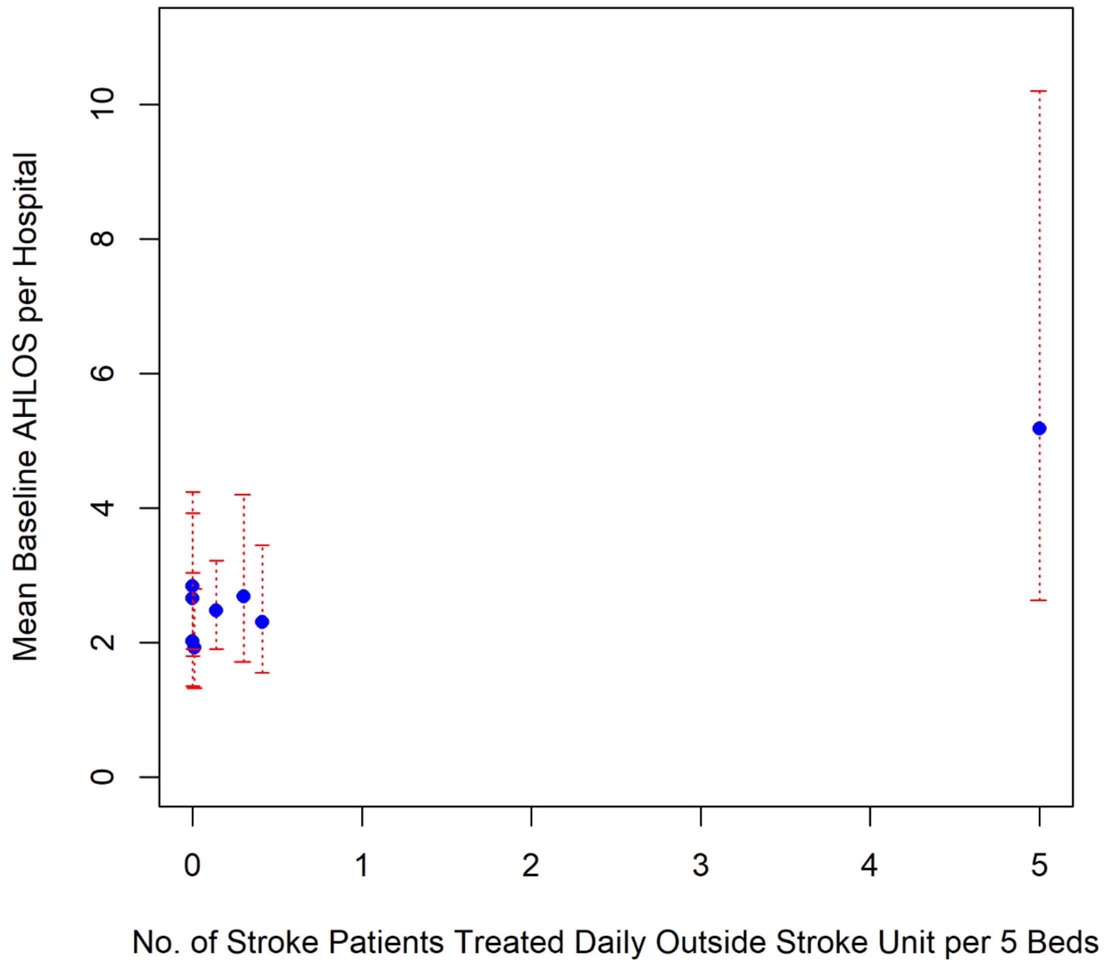

Figure S1 Model estimates of mean baseline acute hospital length of stay (AHLOS) per hospital (in days) and number of stroke patients treated outside the stroke unit per day per five stroke unit beds with 95% confidence intervals. Multiple regression model was adjusted for patient covariates that had a p-value<0.3 in univariable analysis.

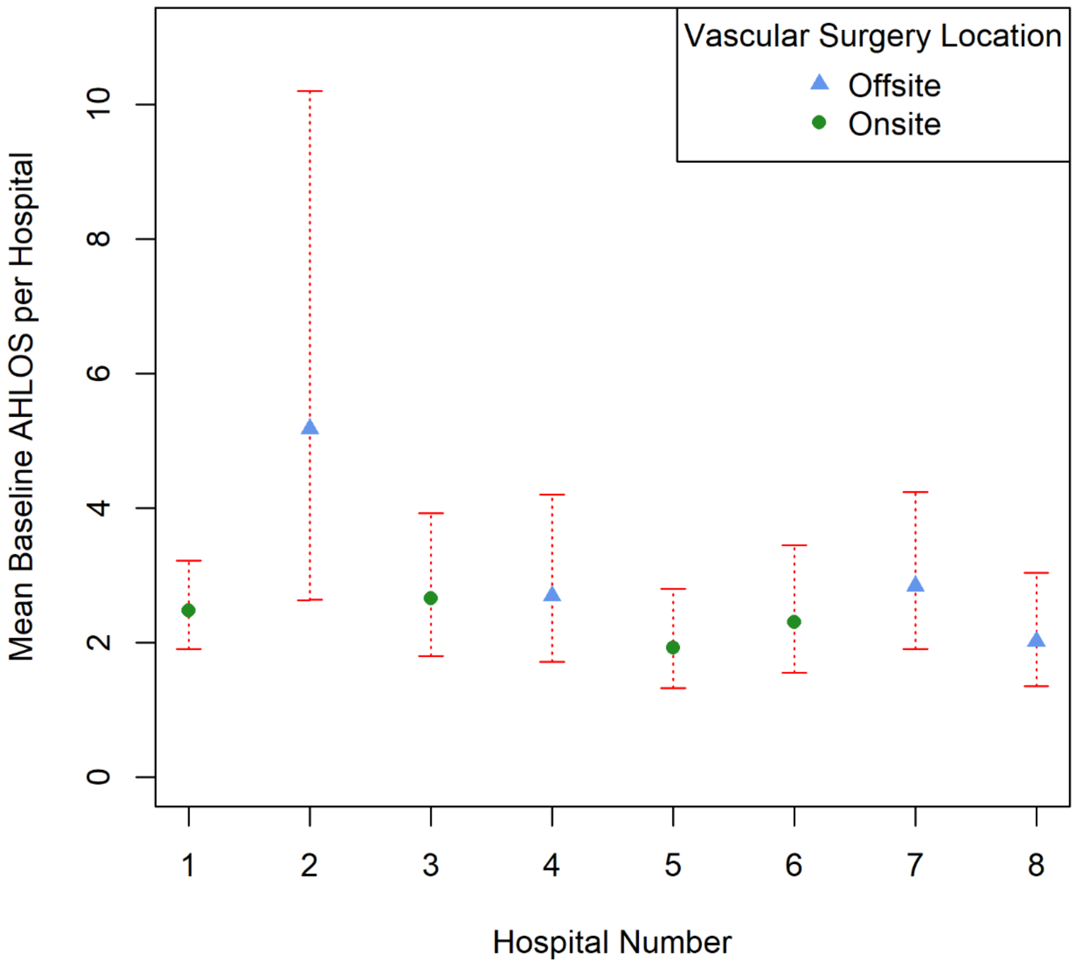

Figure S2 Model estimates of mean baseline acute hospital length of stay (AHLOS) per hospital (in days) and presence of vascular surgery onsite with 95% confidence intervals. Multiple regression model was adjusted for patient covariates that had a p-value<0.3 in univariable analysis.

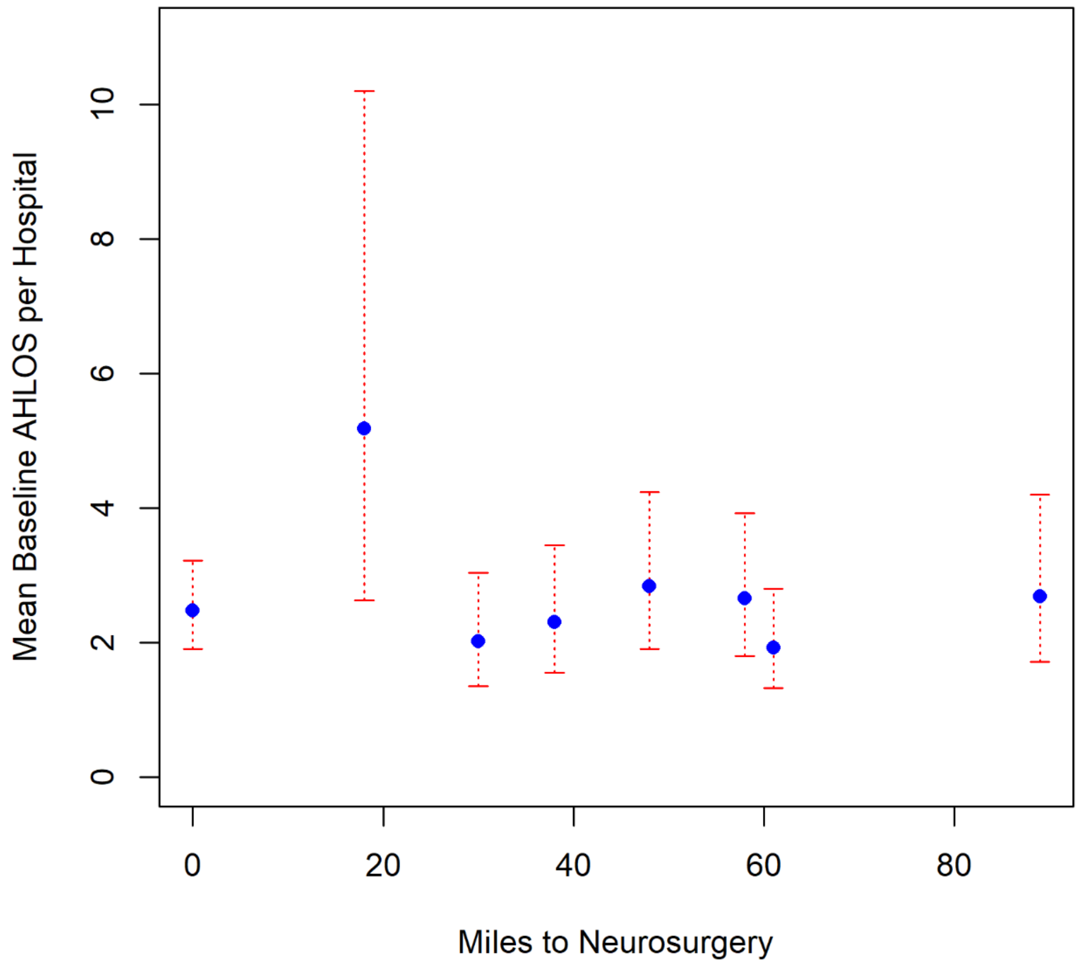

Figure S3 Model estimates of mean baseline acute hospital length of stay (AHLOS) per hospital (in days) and distance to neurosurgical facility with 95% confidence intervals. Multiple regression model was adjusted for patient covariates that had a p-value<0.3 in univariable analysis.

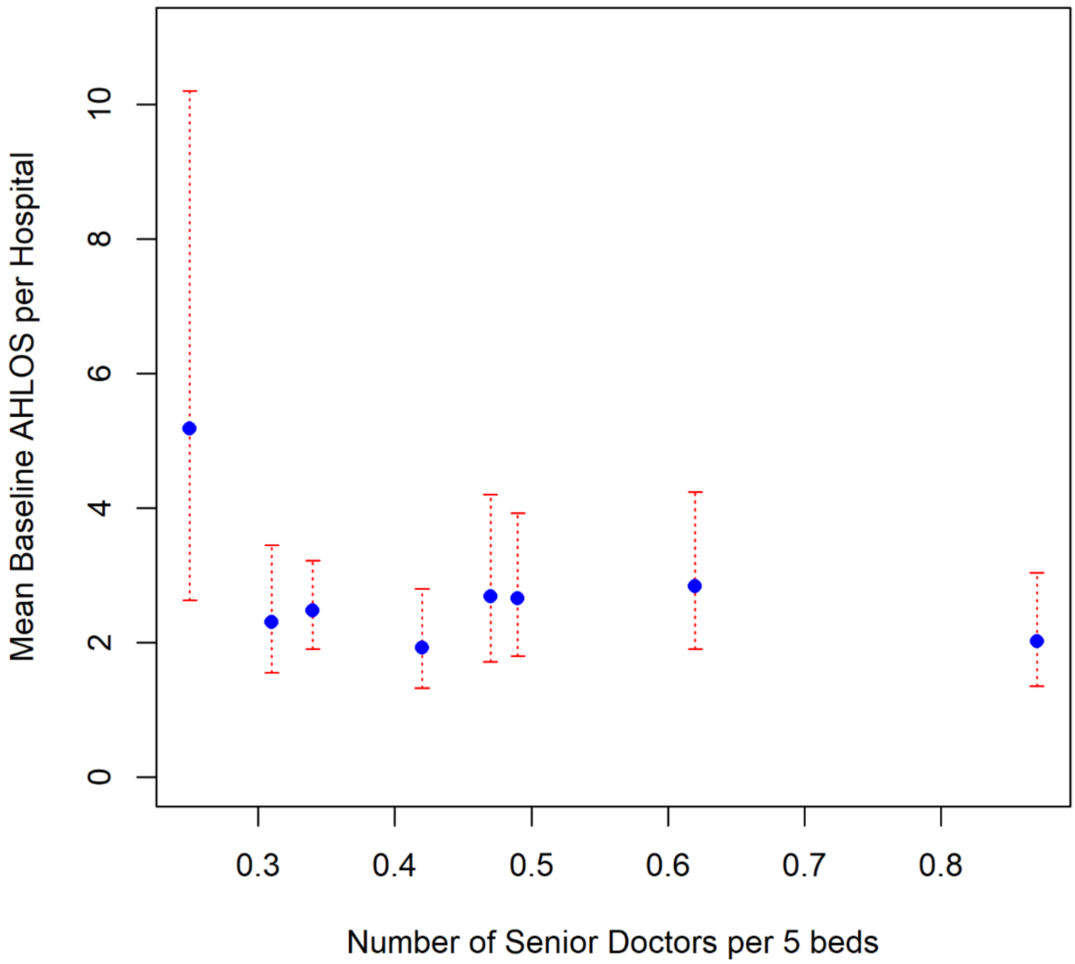

Figure S4 Model estimates of mean baseline acute hospital length of stay (AHLOS) per hospital (in days) and number of fte senior doctors per five beds available during weekdays with 95% confidence intervals. Multiple regression model was adjusted for patient covariates that had a p-value<0.3 in univariable analysis.

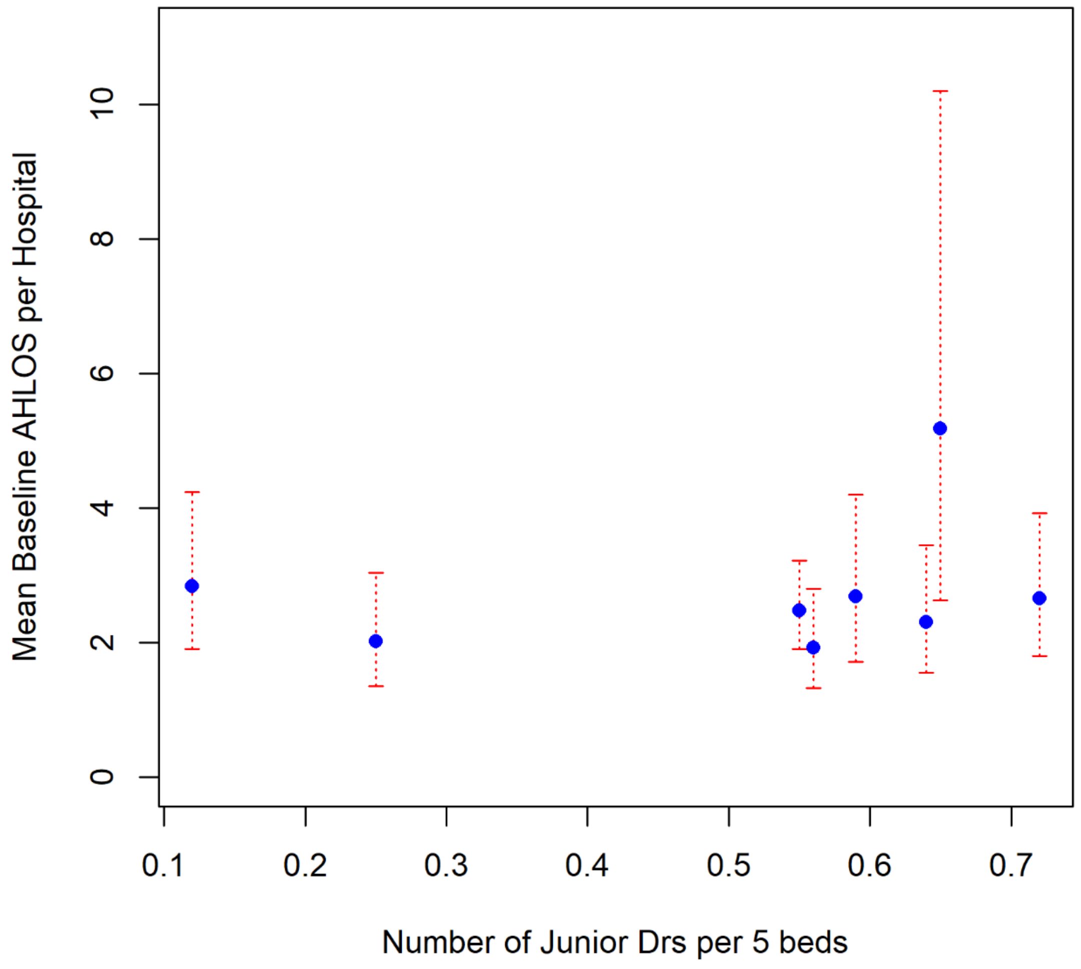

Figure S5 Model estimates of mean baseline acute hospital length of stay (AHLOS) per hospital (in days) and number of fte junior doctors per five beds available during weekdays with 95% confidence intervals. Multiple regression model was adjusted for patient covariates that had a p-value<0.3 in univariable analysis.

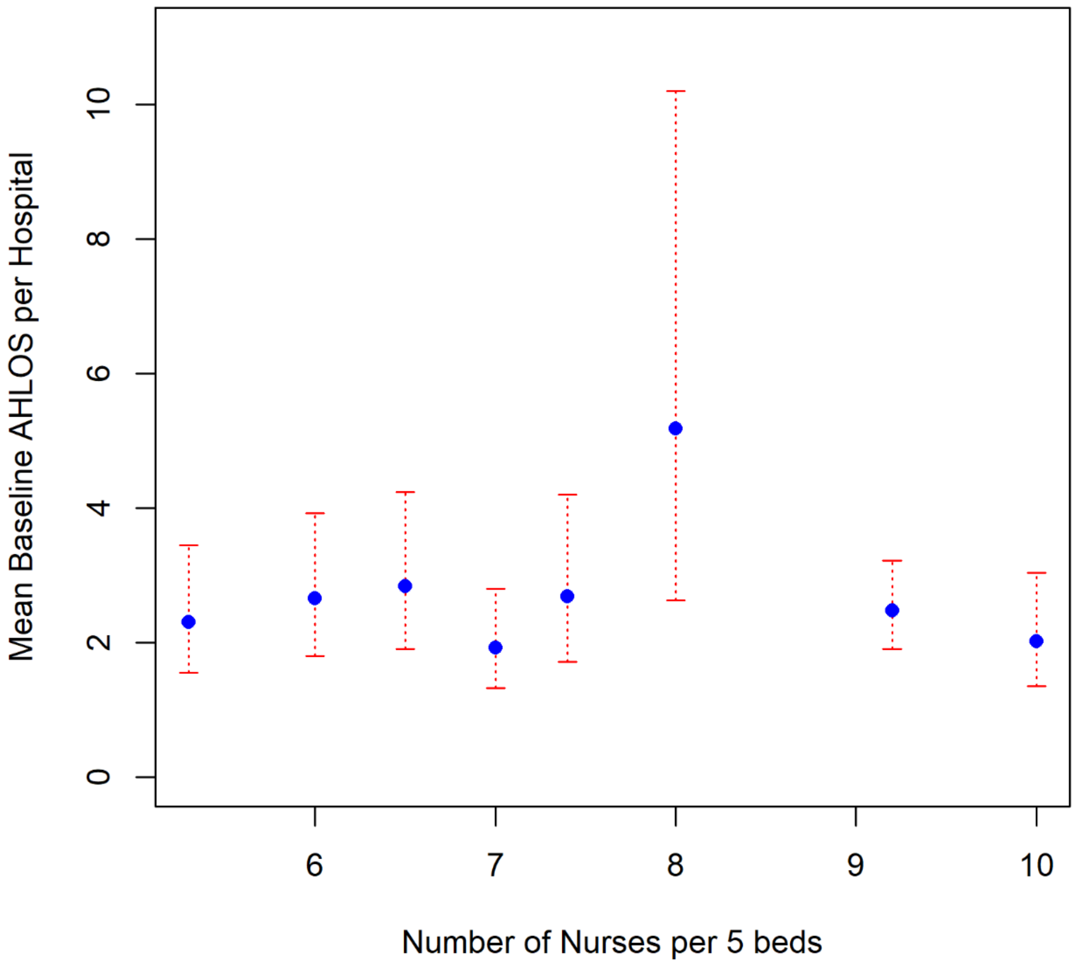

Figure S6 Model estimates of mean baseline acute hospital length of stay (AHLOS) per hospital (in days) and number of fte health care associates and nurses per five beds with 95% confidence intervals. Multiple regression model was adjusted for patient covariates that had a p-value<0.3 in univariable analysis.

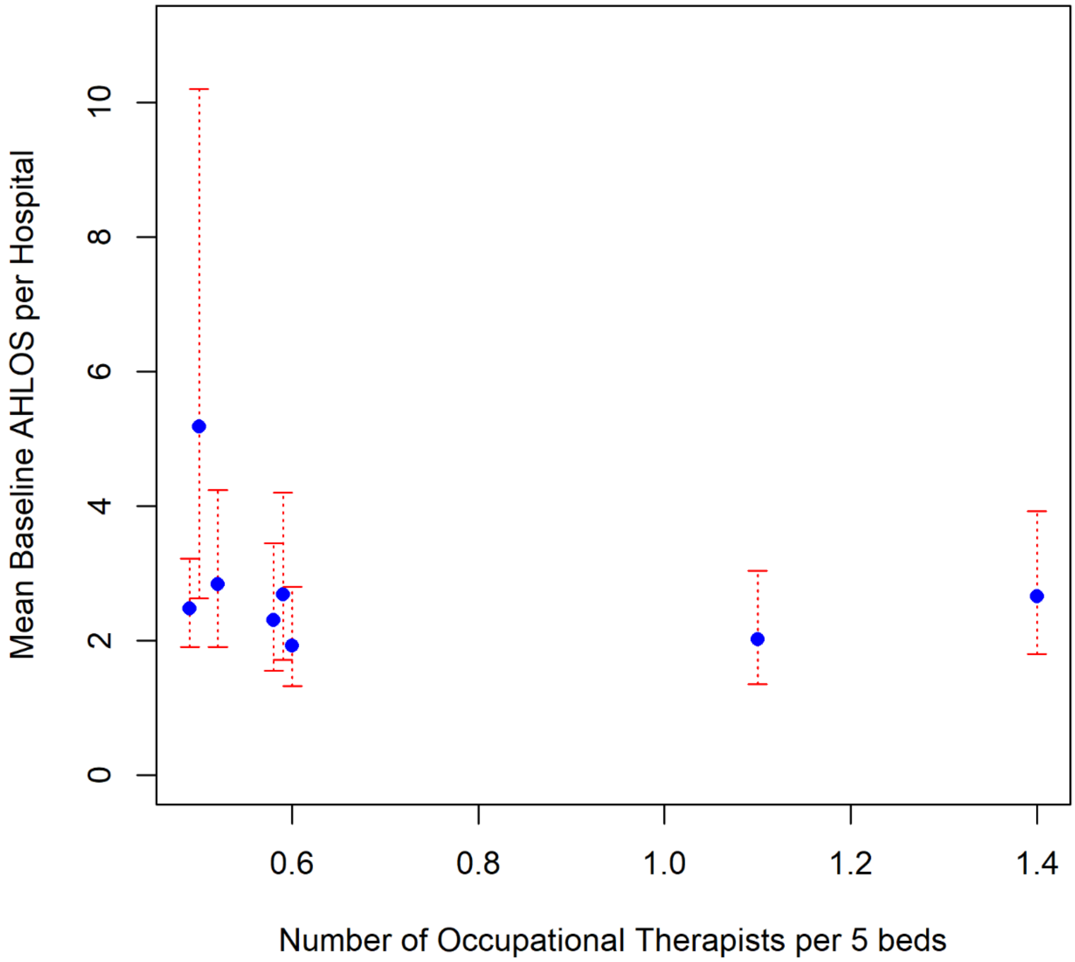

Figure S7 Model estimates of mean baseline acute hospital length of stay (AHLOS) per hospital (in days) and number of fte occupational therapists per five beds with 95% confidence intervals. Multiple regression model was adjusted for patient covariates that had a p-value<0.3 in univariable analysis.

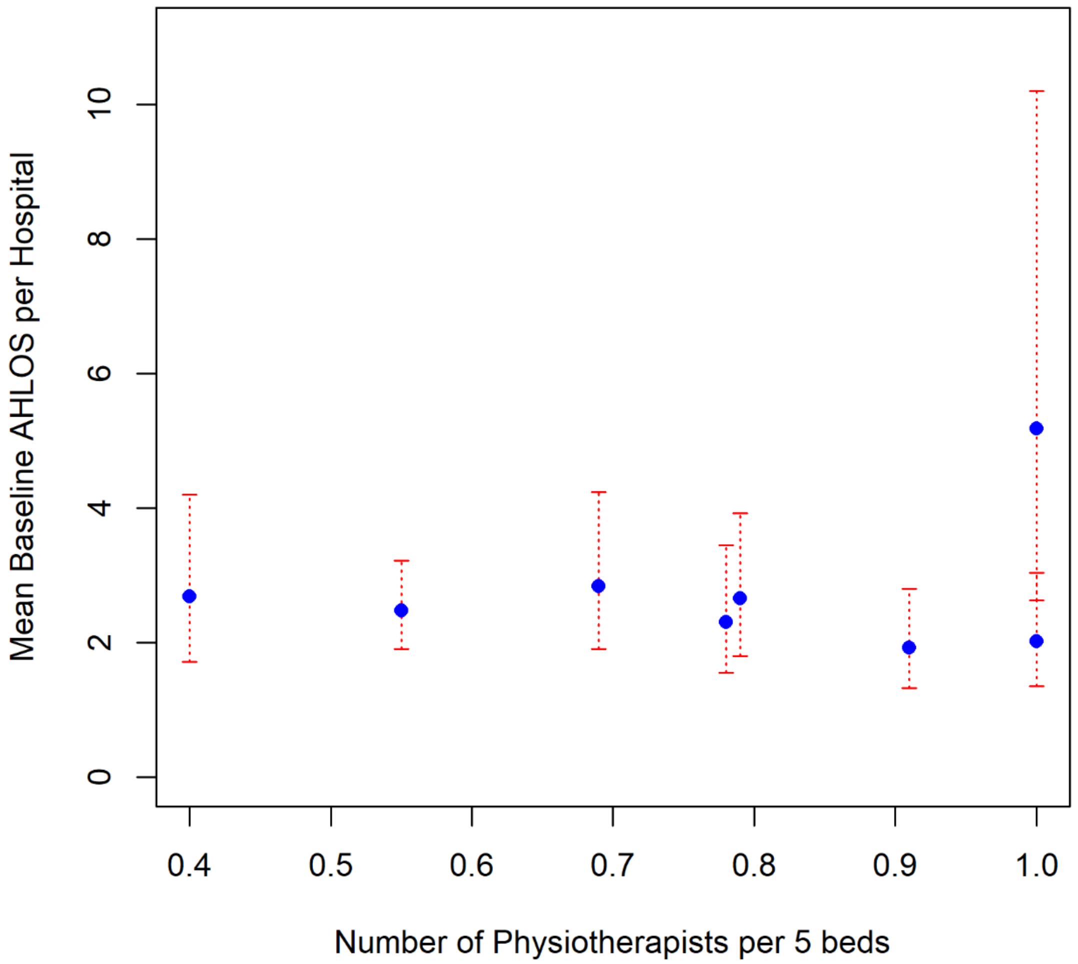

Figure S8 Model estimates of mean baseline acute hospital length of stay (AHLOS) per hospital (in days) and number of fte physiotherapists per five beds with 95% confidence intervals. Multiple regression model was adjusted for patient covariates that had a p-value<0.3 in univariable analysis.

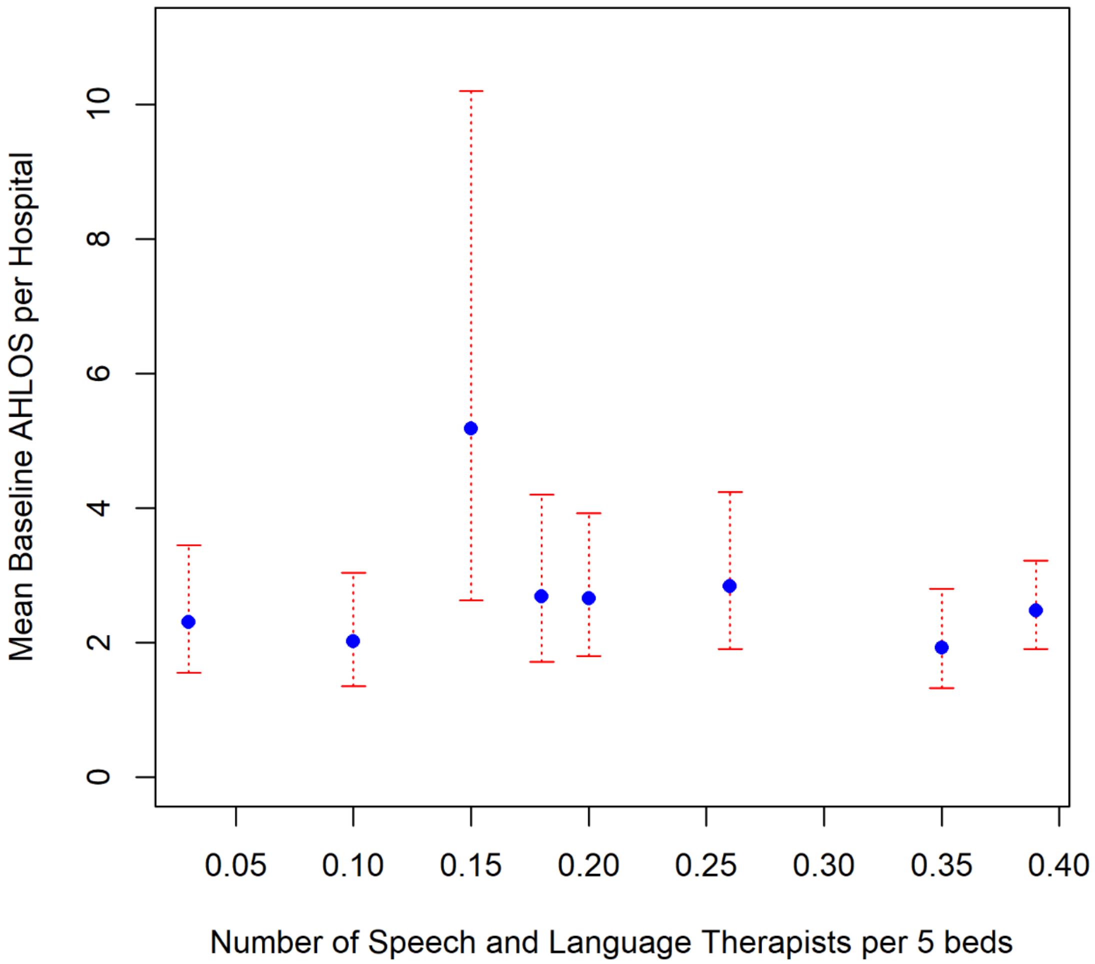

Figure S9 Model estimates of mean baseline acute hospital length of stay (AHLOS) per hospital (in days) and number of fte speech and language therapists per five beds with 95% confidence intervals. Multiple regression model was adjusted for patient covariates that had a p-value<0.3 in univariable analysis.

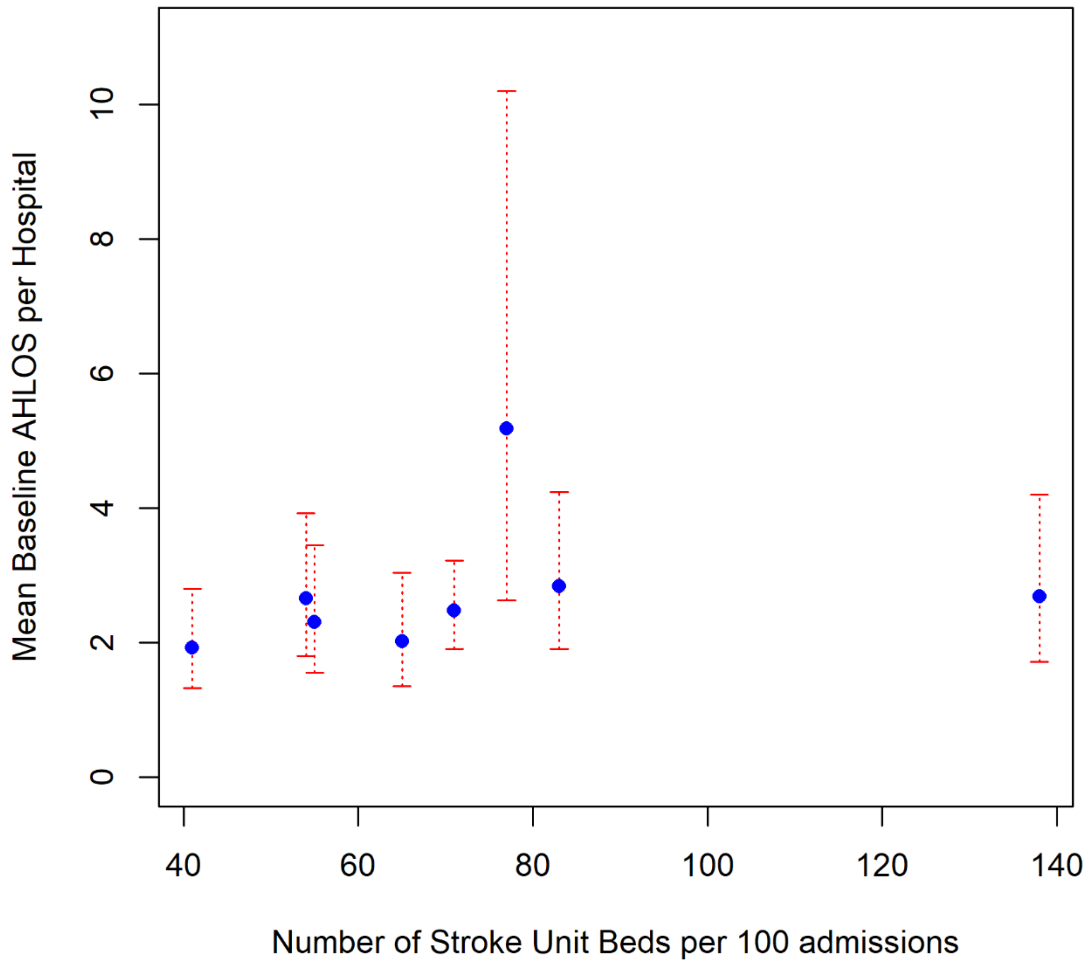

Figure S10 Model estimates of mean baseline acute hospital length of stay (AHLOS) per hospital (in days) and number of total beds present on stroke unit per 100 admissions with 95% confidence intervals. Multiple regression model was adjusted for patient covariates that had a p-value<0.3 in univariable analysis.

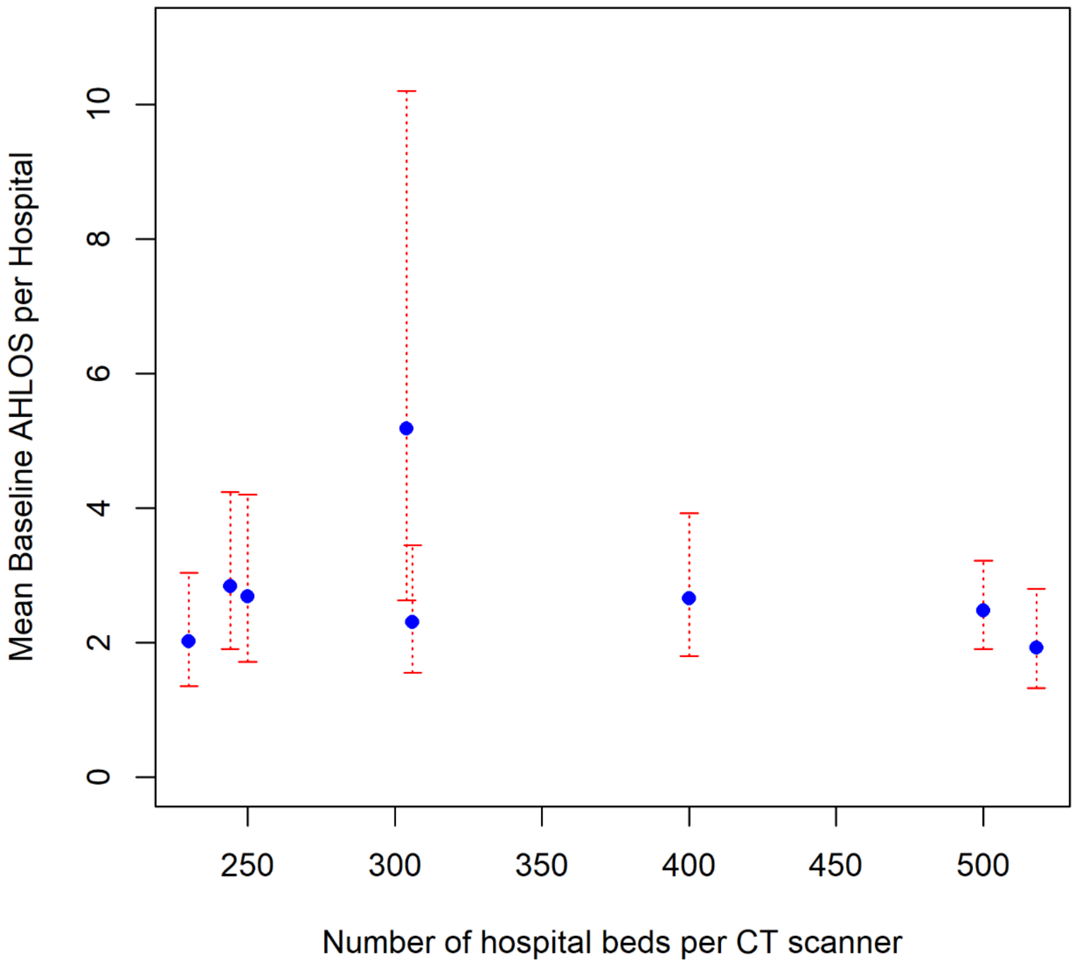

Figure S11 Model estimates of mean baseline acute hospital length of stay (AHLOS) per hospital (in days) and number of hospital beds per computed tomography (CT) scanner with 95% confidence intervals. Multiple regression model was adjusted for patient covariates that had a p-value<0.3 in univariable analysis.

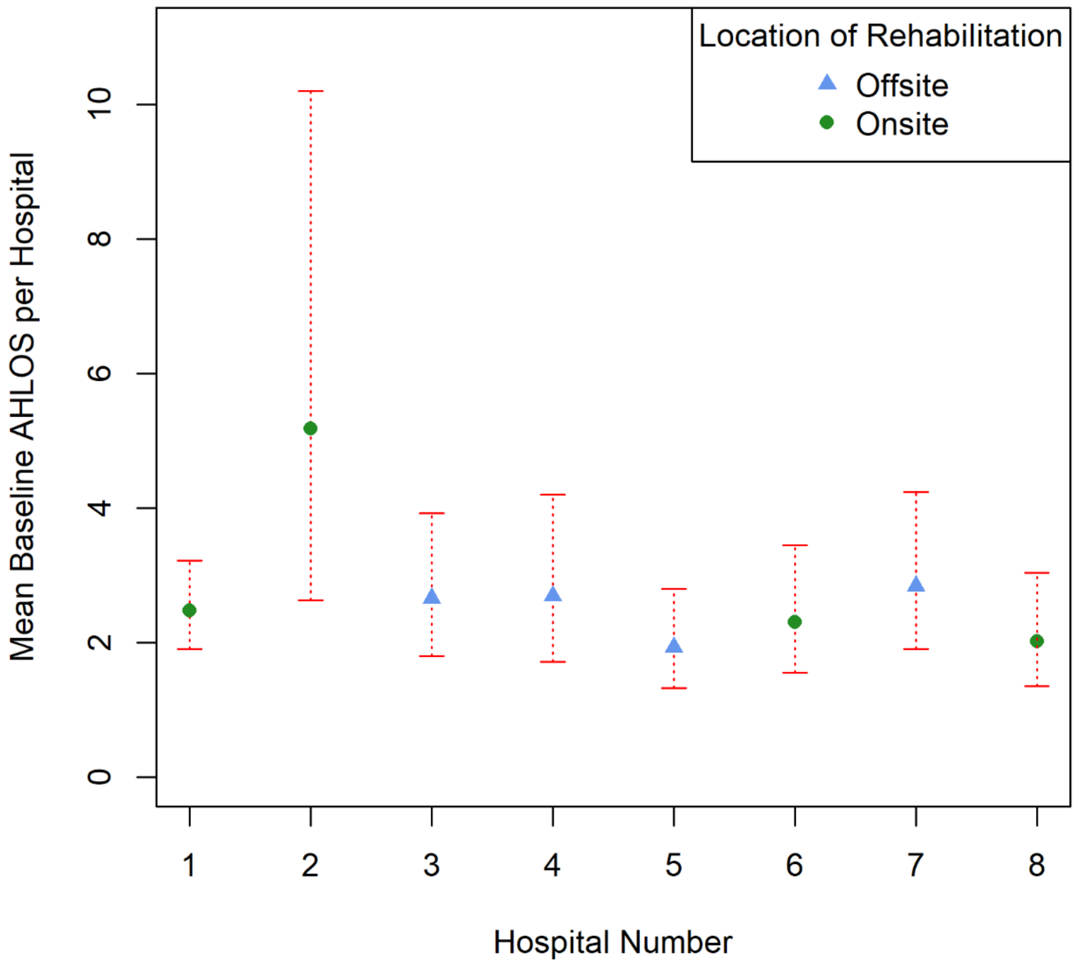

Figure S12 Model estimates of mean baseline acute hospital length of stay (AHLOS) per hospital (in days) and provision of onsite rehabilitation service with 95% confidence intervals. Multiple regression model was adjusted for patient covariates that had a p-value<0.3 in univariable analysis.

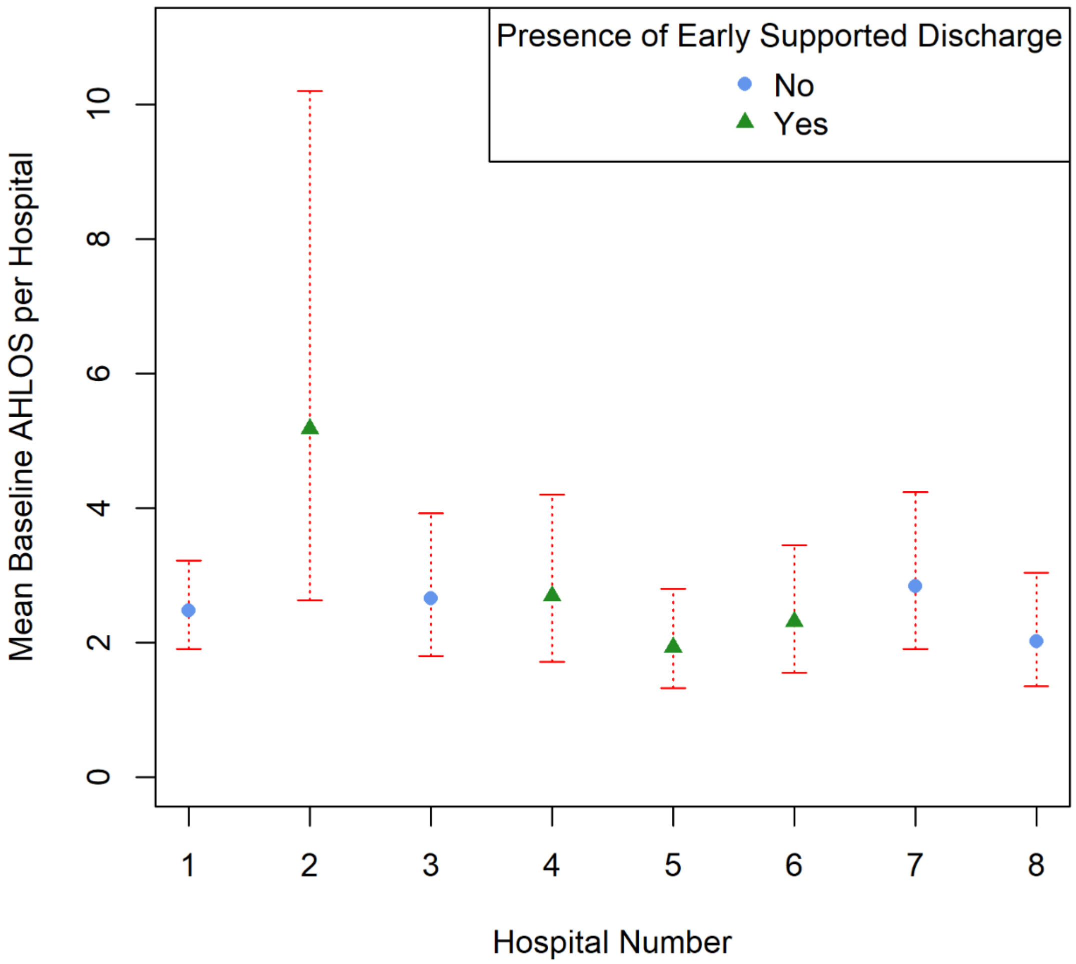

Figure S13 Model estimates of mean baseline acute hospital length of stay (AHLOS) per hospital (in days) and presence of early supported discharge scheme with 95% confidence intervals. Multiple regression model was adjusted for patient covariates that had a p-value<0.3 in univariable analysis.

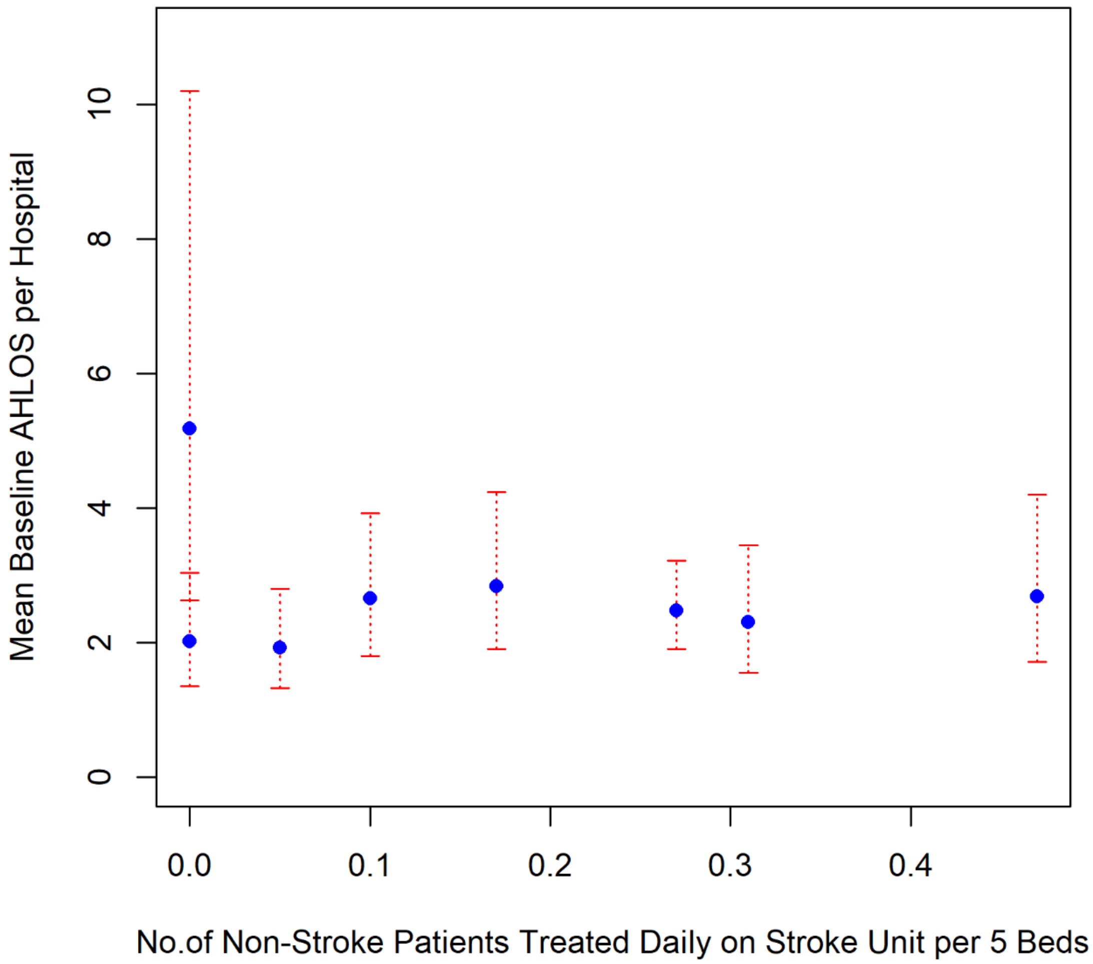

Figure S14 Model estimates of mean baseline acute hospital length of stay (AHLOS) per hospital (in days) and number of non-stroke patients present on the stroke unit per day per five stroke unit beds with 95% confidence intervals. Multiple regression model was adjusted for patient covariates that had a p-value<0.3 in univariable analysis.

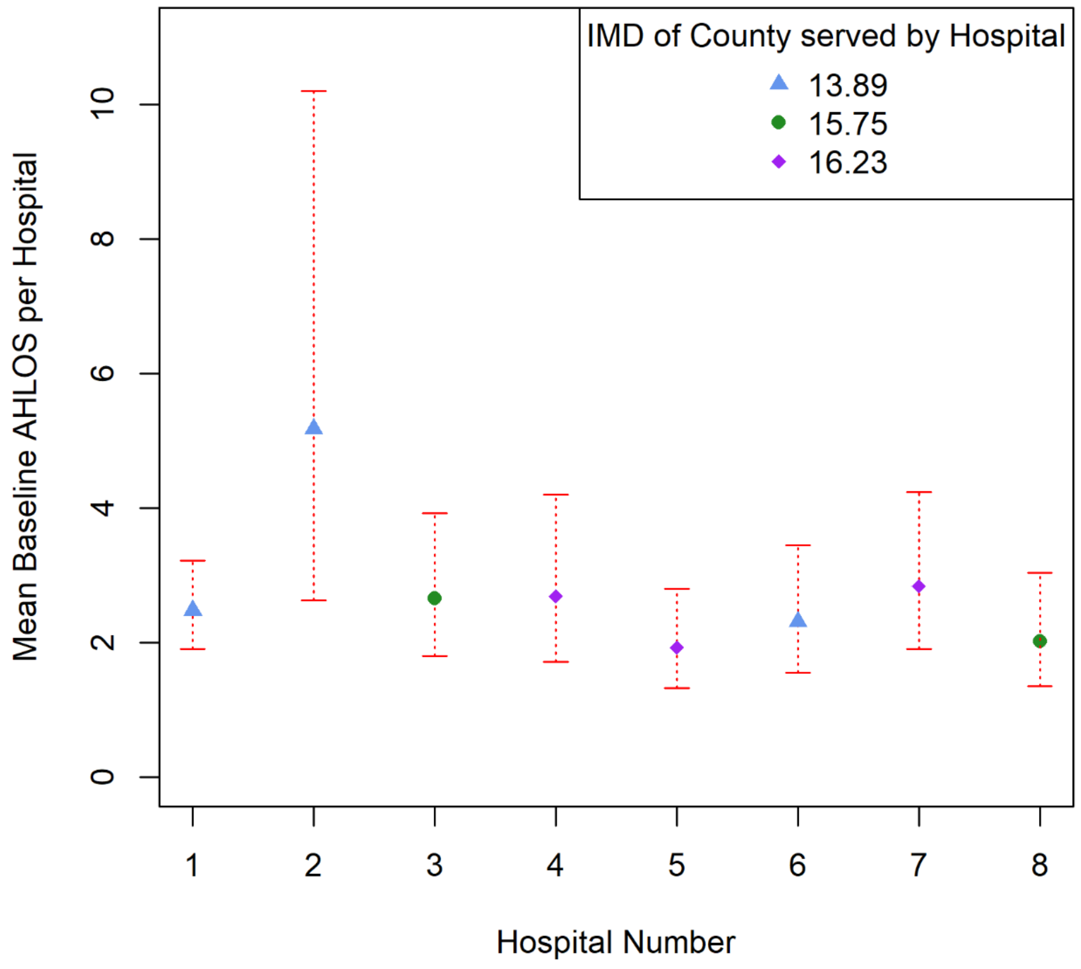

Figure S15 Model estimates of mean baseline acute hospital length of stay (AHLOS) per hospital (in days) and mean Index of Multiple Deprivation (IMD) score of the counties in which the hospital serves with 95% confidence intervals. Multiple regression model was adjusted for patient covariates that had a p-value<0.3 in univariable analysis.
